# Field-Scale Evaluation of Botanical Extracts Effect on the Yield, Chemical Composition and Antioxidant Activity of Celeriac (*Apium graveolens* L. Var. *rapaceum*)

**DOI:** 10.3390/molecules25184212

**Published:** 2020-09-14

**Authors:** Katarzyna Godlewska, Paweł Pacyga, Izabela Michalak, Anita Biesiada, Antoni Szumny, Natalia Pachura, Urszula Piszcz

**Affiliations:** 1 Department of Horticulture, Faculty of Life Sciences and Technology, Wrocław University of Environmental and Life Sciences, 50-363 Wrocław, Poland; anita.biesiada@upwr.edu.pl; 2 Department of Mechanics, Machines and Energy Processes, Faculty of Mechanical and Power Engineering, Wrocław University of Science and Technology, 50-370 Wrocław, Poland; pawel.pacyga@pwr.edu.pl; 3 Department of Advanced Material Technologies, Faculty of Chemistry, Wrocław University of Science and Technology, 50-372 Wrocław, Poland; izabela.michalak@pwr.edu.pl; 4 Department of Chemistry, Faculty of Biotechnology and Food Science, Wrocław University of Environmental and Life Sciences, 50-375 Wrocław, Poland; antoni.szumny@upwr.edu.pl (A.S.); natalia.pachura@upwr.edu.pl (N.P.); 5 Department of Plant Nutrition, Faculty of Life Sciences and Technology, Wrocław University of Environmental and Life Sciences, 50-357 Wrocław, Poland; urszula.piszcz@upwr.edu.pl

**Keywords:** higher plants, biostimulants, ultrasound assisted extraction, homogenisation, foliar spray, celeriac

## Abstract

The use of higher plants for the production of plant growth biostimulants is receiving increased attention among scientists, farmers, investors, consumers and regulators. The aim of the present study was to examine the possibility of converting plants commonly occurring in Europe (St. John’s wort, giant goldenrod, common dandelion, red clover, nettle, valerian) into valuable and easy to use bio-products. The biostimulating activity of botanical extracts and their effect on the chemical composition of celeriac were identified. Plant-based extracts, obtained by ultrasound-assisted extraction and mechanical homogenisation, were tested in field trials. It was found that the obtained formulations increased the total yield of leaves rosettes and roots, the dry weight of leaves rosettes and roots, the content of chlorophyll *a* + *b* and carotenoids, the greenness index of leaves, the content of vitamin C in leaves and roots. They mostly decreased the content of polyphenols and antioxidant activities in leaves but increased them in roots and conversely affected the nitrates content. Extracts showed a varied impact on the content of micro and macroelements, as well as the composition of volatile compounds and fatty acids in the celeriac biomass. Due to the modulatory properties of the tested products, they may be used successfully in sustainable horticulture.

## 1. Introduction

One of the greatest issues confronting the global agriculture is how to ensure the sustainable production of sufficient amounts of food, fibre and bioenergy to fulfil the needs of a growing world population that is forecast to reach 10 billion by 2050 [1,2]. Therefore, to achieve this aim, whilst maintaining actual prices, the crop yields should be enhanced by 1.1–1.2% annually with reference to the levels in 2010 [3]. Primarily, the use of mineral fertilisers (especially rich in nitrogen—N) has allowed this goal to be achieved [2,4]. However, a steadily increasing demand for synthetic fertilisers has been observed over a few decades. The worldwide consumption of NPK fertilisers is expected to rise from 135.4 million tonnes (Mt) in 2000/2001 to 204 Mt in 2023/2024 [5]. Nevertheless, the fertilisation effectiveness remains comparatively low, which is caused by the fact that only 50–60% of N [5,6] and K and 10–25% of P are estimated to be utilised by cultivated plants [5]. Taking this into account, the global demand for N has been expected to reach 107 teragrams (Tg) N per year (with a limit of 50 Tg N reactive not absorbed by plants) by the year 2050 [2]. This low fertilisation results in numerous adverse effects on plant metabolism. The deficiency of N, P and K causes changes in intracellular pH, ionic disbalance, protein, organic acids and carbohydrates content. The lack of potassium increases plant sensitivity to oxidative stress and leads to chloroplast degradation and increase in enzyme antioxidative activity. The insufficiency of N has a negative impact on the chlorophyll content and photosynthetic activity thereby reducing the growth and yield of crops. The deficiency of phosphorus induces the increase in H_2_O_2_ concentration affecting the cell redox balance due to the increased content of antioxidative enzymes [7]. On the other hand, a higher fertilisation efficiency can be achieved by using slow/controlled release of nitrogen fertilisers that reduce the loss of N through leaching and evaporation [5]. Furthermore, the growing dependence on N fertilisers is a major issue in prevailing agriculture practices and has adverse environmental impacts due to several side effects, namely high costs of input and production [3,4], the contribution to the emission of greenhouse gases (up to 70% of the worldwide N_2_O) [8] and water eutrophication [6] which is the major threat to biodiversity [4]. Nitrogen is a crucial nutrient for the proper growth of crops and is a restrictive agent for food production [2] so it stands to reason that there is a need to seek a solution to enhance the efficiency of N fertilisers utilisation [9] without negative effects on the natural environment [6].

In recent decades, the genetic selection and the development of new cultivars with increased ability to absorb nutrients and resistance to abiotic (e.g., soil composition, salinity, acidity, temperatures, drought, humidity, pollution, rain, wind, ultraviolent radiation) and biotic (e.g., bacteria, fungi, viruses, herbivores) factors has been proposed as a cost-effective and sustainable solution providing high yield of crops. Notwithstanding, over the past 10 years, interest in biostimulants has been rising amongst researchers, experts, industries, and producers. These eco-friendly products can provide enhanced crop performance, yield stability [10] and quality, nutrient uptake, plant tolerance to abiotic and biotic stresses, activity of rhizosphere microbes and soil enzymes, production of hormones and growth regulators in soil and plants, photosynthetic process [11,12], and extended shelf life and storage life of fruits [12]. Higher yields without suspending the ecological practices are the main advantages of these products [12]. It should be noted that biostimulants exhibit their activity at low concentrations [7]. These bioproducts do not supply plants with nutrients and cannot be defined as fertilisers—they can be treated as additives to fertilisers [12]. Biostimulants can be also used in seed treatment [13], as soil additives in the form of solutions, granules, powders (affect the structure of roots and increase the absorption of nutrients), as a foliar spray (protect crops from stress) or additives to hydroponic solutions (taken up by plants along with water) [12]. Biostimulants can be used on a regular basis in the vegetative stage or preventively before or during stress and loss of plant’s vital forces [12]. They are produced mostly from raw materials rich in bioactive compounds which show multiple modes of action (additive and/or synergistic) that still remain unidentified [11] and might involve the activation of N metabolism or P release from soils, generic stimulation of soil microbial activity or stimulation of root growth and enhanced plant establishment [13]. Biostimulants exhibit complex composition which may consist of amino acids, peptides, proteins, betaines, sugars, aminopolysaccharides, lipids, vitamins, nucleotides or nucleosides, humic substances, beneficial elements, phenolic compounds, furostanol glycosides, sterols, and plant hormones or hormone-like substances [13] such as auxins, gibberellins, cytokines and triacontanol correlated with the positive impact on plant growth [11].

Within this study, we decided to conduct the field trials to evaluate the impact of innovative botanical extracts on the yield, chemical composition and antioxidant activity of celeriac (*Apium graveolens* L. var. *rapaceum*). The raw materials (herb of St. John’s wort, leaves of giant goldenrod, flowers and leaves of common dandelion, flowers of red clover, leaves of nettle and roots of valerian) were chosen based on our previous studies conducted under laboratory conditions [14,15]. Celery root was chosen as a model plant because both roots and leaves are edible, are low in calories and carbohydrates and can be consumed fresh or processed [16]. It is an aromatic plant with important pharmaceutical properties [17]. Celeriac is a rich source of vitamins C and K, iron, manganese, potassium, phosphorus, magnesium and foliate [16,18,19] and exhibits antiphlogistic, antimicrobial, antiviral, antiallergic and antioxidant activity [16]. The essential oils are used as a curative for skin problems and also in rheumatism. They also show the calming effect and are used as a diuretic [19]. This vegetable is cultivated globally, for example in 2017 the European Union produced 526 thousand tonnes of it [17]. In Poland, its production occupies some 4–6 thousand ha and the harvest reaches approximately 110 thousand tonnes [20]. This vegetable has high water and nutrient requirements, especially for nitrogen—the optimum dose is 200 kg·N ha^−1^ [21].

The future research of plant biostimulants should bridge the gap between the laboratory data (on single preparations) and field trials (on mixtures often combined with fertilisers). The research hypothesis assumes that plant extracts applied in field conditions affect plant growth parameters (e.g., fresh and dry weight), their chemical composition (e.g., photosynthetic pigments, vitamin C, total phenolic compounds, nitrates, volatile compounds, fatty acids) and antioxidant activity.

## 2. Results

### 2.1. Total Yield, Fresh and Dry Weight of Leaves Rosette and Roots

**Figure 1:**
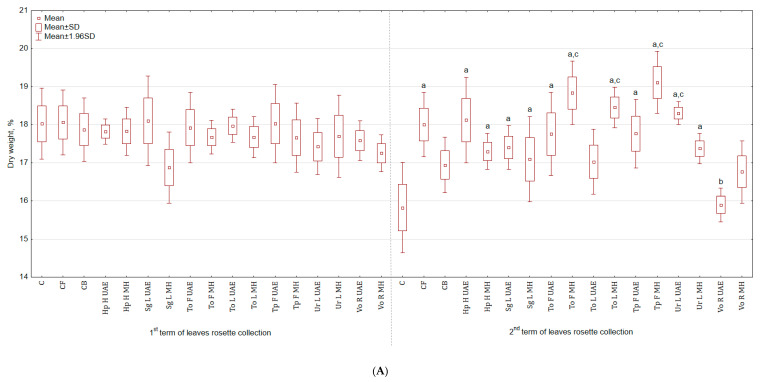

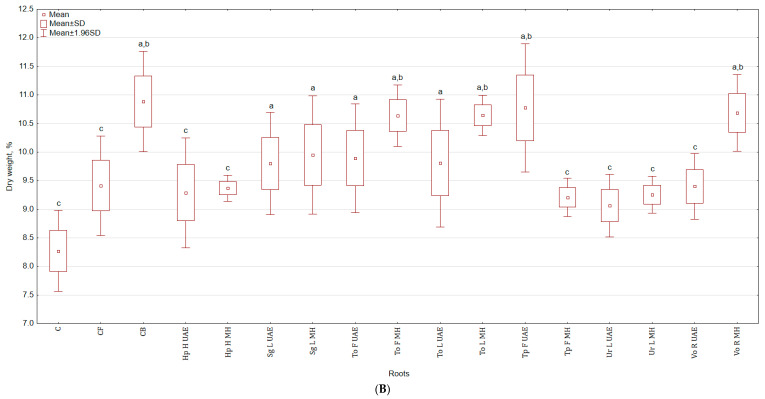
(**A**) Effect of the foliar application of botanical extracts on the dry weight of celeriac leaves rosette (N = 3). (a) Statistically significant differences (*p* < 0.05) between the control group (C) and extracts. (b) Statistically significant differences (*p* < 0.05) between water formulation (CF) and extracts. (c) Statistically significant differences (*p* < 0.05) between commercial biostimulant (CB) and extracts. 1st term of leaves rosette collection—samples taken for analyses 7 days after the second spraying. 2 term of leaves rosette collection—samples taken for analyses after harvesting. Abbreviations: UAE, ultrasound assisted extraction; MH, mechanical homogenisation; Hp H, *Hypericum perforatum* L. (St. John’s wort, herb); Sg L, *Solidago gigantea* Ait. (giant goldenrod, leaf); To F, To L, *Taraxacum officinale* (L.) Weber ex F.H. Wigg (common dandelion, flower, leaf); Tp F, *Trifolium pratense* L. (red clover, flower); Ur L, *Urtica dioica* L. (nettle, leaf); Vo R, *Valeriana officinalis* L. (valerian, root). (**B**) Effect of the foliar application of botanical extracts on the dry weight of celeriac roots (N = 3).

The application of botanical extracts had a varied impact on the total yield of celeriac (Table 1). For instance, in the group treated with Hp H MH, the yield of leaves rosette was higher by 43.7% and 66.2% than in C and CB, respectively and for roots by 68.2% and 52.0%. The lowest yield was observed in the group sprayed with Vo R MH—leaves rosette yield was by 42.6% and 33.6% lower, while roots by 48.9% and 53.9% lower than in C and CB, respectively. The fresh weight of celeriac (Table 1), in roots size range < 5 cm, increased in the group treated with Tp F UAE by 29.9% (in relation to C) and by 86.6% (in relation to CB) for roots and with To F UAE by 152.4% and 104.9% for leaves rosette compared with C and CB, respectively. The least stimulating effects were noted after application of Sg L MH extracts—fresh weight of leaves rosette was higher by 2.8% and 21.1%, while roots by 48.4% and 25.9% when compared with C and CB, respectively. Extracts did not stimulate the weight of plants in roots size range from 5 to 9 cm. Plant extracts promoted the growth of roots wider than 9 cm, while in the control group there were no plants of this size. The non-marketable yield constituted from 0 to 4.8%. There was no significant difference in the dry weight content in leaves rosette harvested in the first term but one was observed in the second term in both above-ground parts and roots (Figure 1A). The foliar application of Tp F MH increased the dry weight of leaves rosette by 20.8% and 12.8% in comparison to C and CB. In the case of roots (Figure 1B), the highest weight was observed for Tp F UAE (30.2% more than in C). The root:shoot ratio was the highest for Tp F UAE and Vo R MH (for both higher by 18.5% than in C but not statistically higher than in CB), while the lowest for Tp F MH and Ur L UAE (lower by 9.3% and 22.2% than in C and CB).

### 2.2. The Photosynthetic Pigments, Greenness Index of Leaves, and Leaves Colour

**Figure 2:**
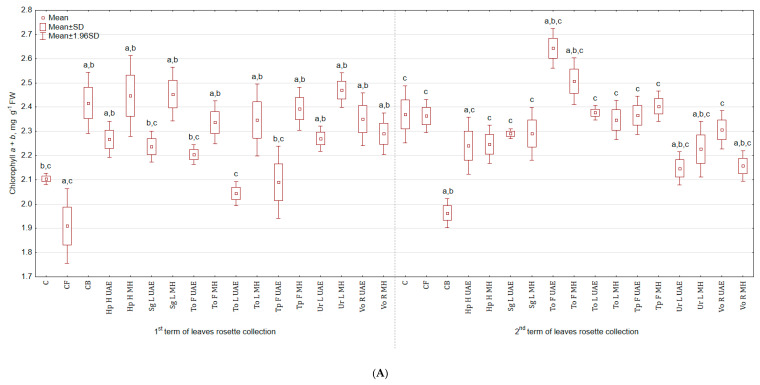

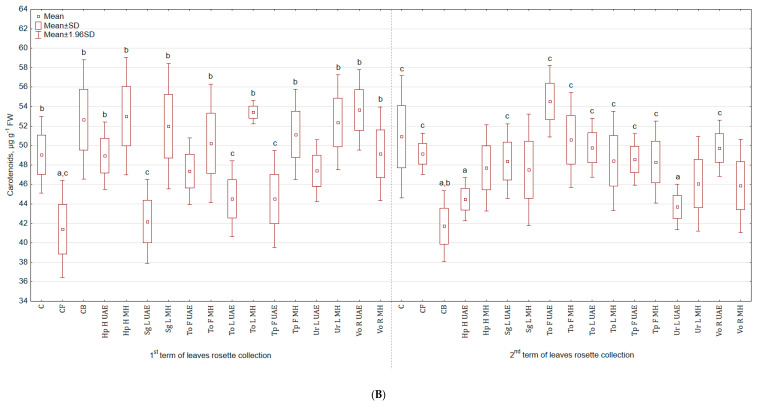

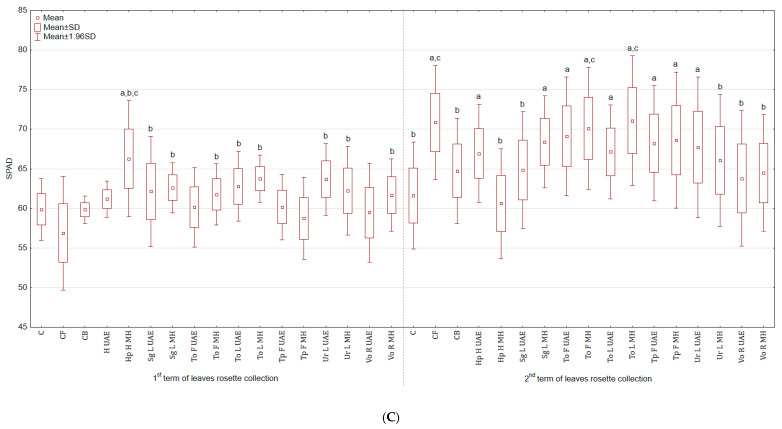
(**A**)**.** Effect of the foliar application of botanical extracts on the chlorophyll *a* + *b* content of celeriac leaves (N = 4). (**B**) Effect of the foliar application of botanical extracts on the carotenoids content of celeriac leaves (N = 4). (**C**) Effect of the foliar application of botanical extracts on the SPAD values of celeriac leaves (N = 10). (a) Statistically significant differences (*p* < 0.05) between the control group (C) and extracts. (b) Statistically significant differences (*p* < 0.05) between water formulation (CF) and extracts. (c) Statistically significant differences (*p* < 0.05) between commercial biostimulant (CB) and extracts. 1st term of leaves rosette collection—samples taken for analyses 7 days after the second spraying. 2nd term of leaves rosette collection—samples taken for analyses after harvesting. Abbreviations: UAE, ultrasound assisted extraction; MH, mechanical homogenisation; Hp H, *Hypericum perforatum* L. (St. John’s wort, herb); Sg L, *Solidago gigantea* Ait. (giant goldenrod, leaf); To F, To L, *Taraxacum officinale* (L.) Weber ex F.H. Wigg (common dandelion, flower, leaf); Tp F, *Trifolium pratense* L. (red clover, flower); Ur L, *Urtica dioica* L. (nettle, leaf); Vo R, *Valeriana officinalis* L. (valerian, root).

Tested plant-based extracts increased the content of chlorophyll *a* + *b* (Figure 2A), especially after the second foliar application (more by 17.6% for Ur L MH and by 16.7% for both Hp H MH and Sg L MH than in C; there were no significant differences between treatments and CB). After third spraying, the highest content of green pigment was observed in the group treated with To F UAE (11.4% and 34.7% more than in C and CB), while the lowest with Ur L UAE (9.3% less than in C and 9.7% more than in CB). In general, the examined plant extracts did not stimulate the content of carotenoids (Figure 2B) and the colour of leaves (Table 2). The statistically significant enhancement was noted in SPAD values (Figure 2C) after third spraying for To L MH (more by 15.3% and 9.8% than in C and CB) and diminution for Hp H MH (less by 1.6% and 6.4% than in C and CB).

### 2.3. Vitamin C

**Figure 3:**
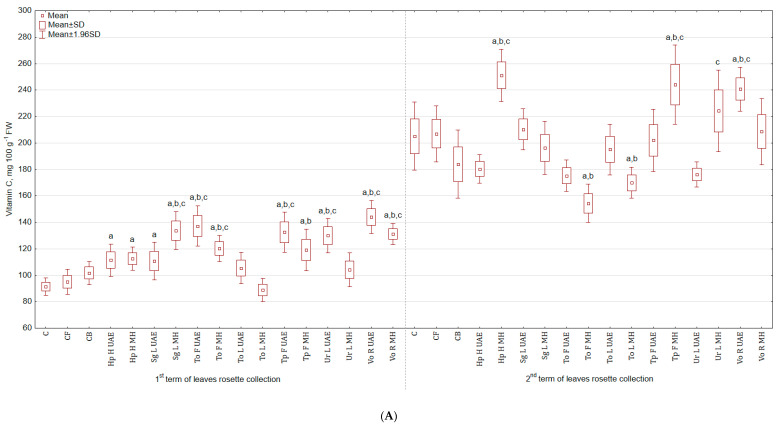

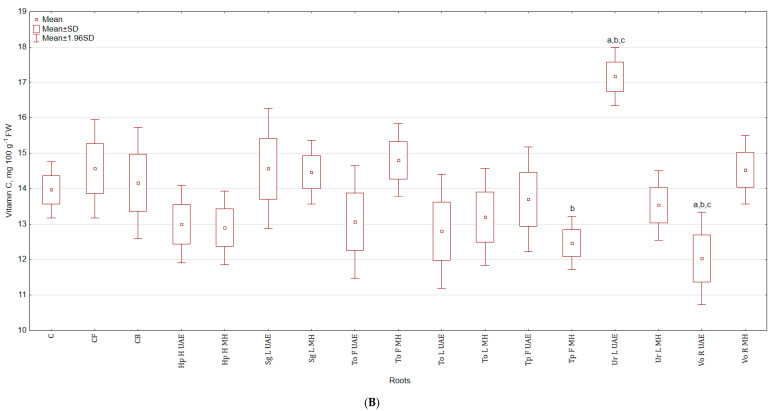
(**A**) Effect of the foliar application of botanical extracts on the vitamin C content of celeriac leaves (N = 4). (**B**) Effect of the foliar application of botanical extracts on the vitamin C content of celeriac roots (N = 4). (a) Statistically significant differences (*p* < 0.05) between the control group (C) and extracts. (b) Statistically significant differences (*p* < 0.05) between water formulation (CF) and extracts. (c) Statistically significant differences (*p* < 0.05) between commercial biostimulant (CB) and extracts. 1st term of leaves rosette collection—samples taken for analyses 7 days after the second spraying. 2nd term of leaves rosette collection—samples taken for analyses after harvesting. Abbreviations: UAE, ultrasound assisted extraction; MH, mechanical homogenisation; Hp H, *Hypericum perforatum* L. (St. John’s wort, herb); Sg L, *Solidago gigantea* Ait. (giant goldenrod, leaf); To F, To L, *Taraxacum officinale* (L.) Weber ex F.H. Wigg (common dandelion, flower, leaf); Tp F, *Trifolium pratense* L. (red clover, flower); Ur L, *Urtica dioica* L. (nettle, leaf); Vo R, *Valeriana officinalis* L. (valerian, root).

The highest content of vitamin C was observed in leaves (Figure 3A), mostly after the second application of extracts. For instance, Vo R UAE increased the content of this vitamin by 57.6% and 41.6% in the first harvest, while Hp H MH increased the content by 22.3% and 36.5% in the second harvest in comparison to C and CB, respectively. The lowest values were observed in leaves collected in the 1st term for To L MH (2.8% and 12.2% less) and for the 2nd term for To F MH (24.7% and 15.8% less). In the case of roots (Figure 3B), the greatest level of vitamin was noted in the group treated with Ur L UAE (22.9% and 21.2% more than in C and CB), while the lowest for Vo R UAE (13.9% and 15.1% less).

### 2.4. Total Phenolic Compounds and Antioxidant Activity (DPPH, ABTS, and FRAP)

**Figure 4:**
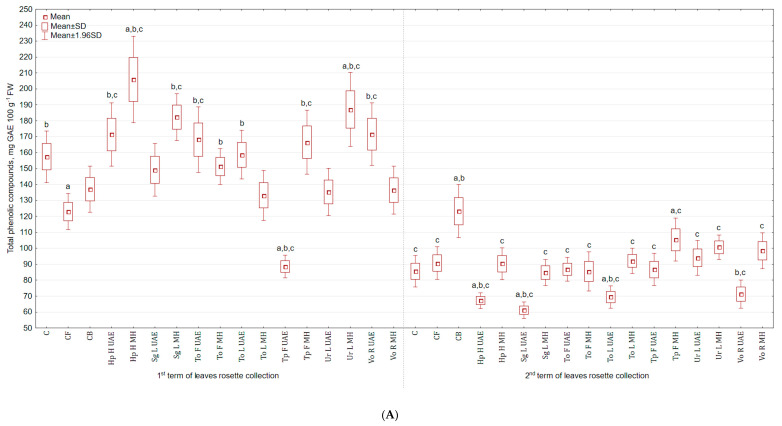

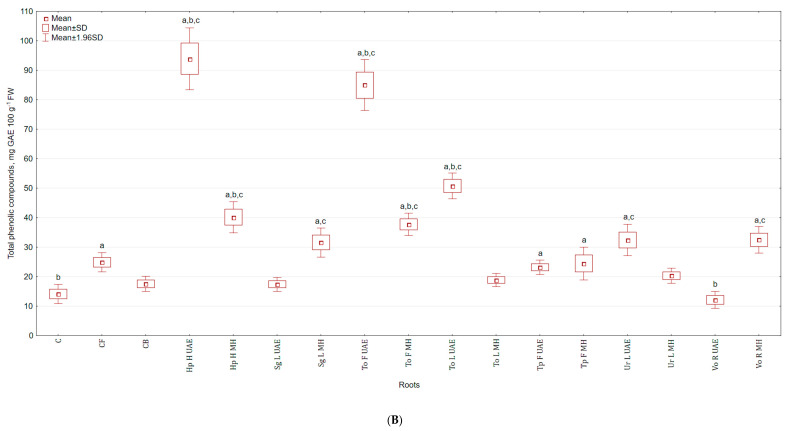
(**A**) Effect of the foliar application of botanical extracts on the total phenolic compounds of celeriac leaves (N = 4). (**B**) Effect of the foliar application of botanical extracts on the total phenolic compounds of celeriac roots (N = 4). (a) Statistically significant differences (*p* < 0.05) between the control group (C) and extracts. (b) Statistically significant differences (*p* < 0.05) between water formulation (CF) and extracts. (c) Statistically significant differences (*p* < 0.05) between commercial biostimulant (CB) and extracts. 1st term of leaves rosette collection—samples taken for analyses 7 days after the second spraying. 2nd term of leaves rosette collection—samples taken for analyses after harvesting. Abbreviations: UAE, ultrasound assisted extraction; MH, mechanical homogenisation; Hp H, *Hypericum perforatum* L. (St. John’s wort, herb); Sg L, *Solidago gigantea* Ait. (giant goldenrod, leaf); To F, To L, *Taraxacum officinale* (L.) Weber ex F.H. Wigg (common dandelion, flower, leaf); Tp F, *Trifolium pratense* L. (red clover, flower); Ur L, *Urtica dioica* L. (nettle, leaf); Vo R, *Valeriana officinalis* L. (valerian, root).

Similar pattern, as for vitamin C, was found for the content of total phenolic compounds (TPC) in the examined celeriac biomass and its antioxidant activities. The highest TPC in leaves collected in the 1st term was achieved for Hp H MH extract (30.8% and 50.2% more than in C and CB), while the lowest for Tp F UAE (43.7% and 35.4% less than in C and CB). For leaves collected in the second term, the application of Tp F MH increased the content of TPC (23.1% more than in C), while Sg L UAE lowered (by 28.6% and 50.4% in comparison to C and CB) (Figure 4A). However, majority of extracts increased the content of polyphenols in roots, e.g., Hp H UAE by 5.7 and 4.3 times more than in C and CB, respectively (Figure 4B). The lowest content of TPC was noted in roots treated with Vo R UAE (14.0% and 31.1% less).

**Figure 5:**
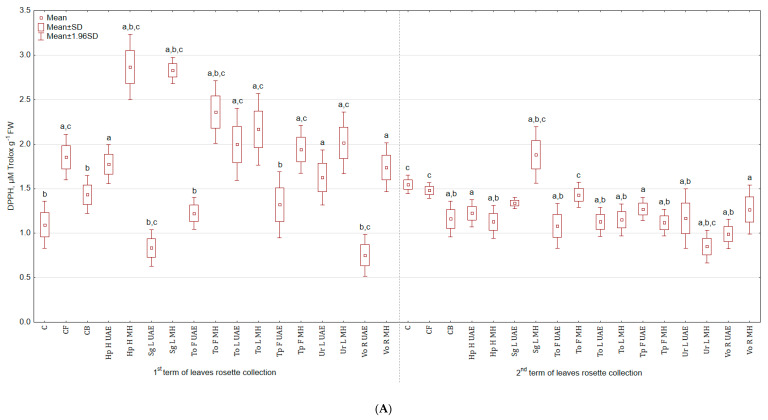

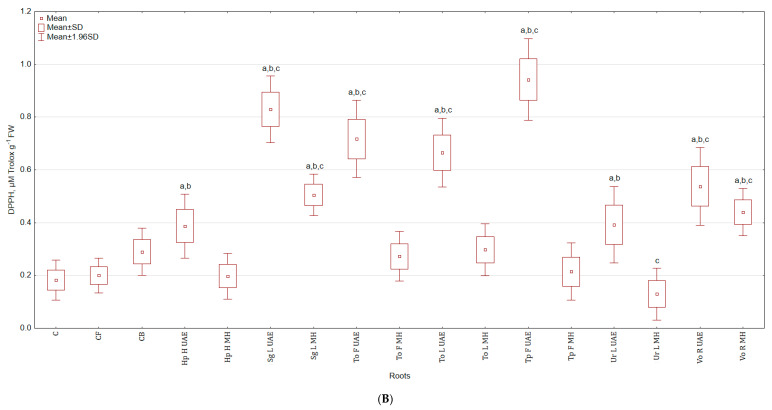
(**A**) Effect of the foliar application of botanical extracts on the antioxidant activity DPPH of celeriac leaves (N = 4). (**B**) Effect of the foliar application of botanical extracts on the antioxidant activity DPPH of celeriac roots (N = 4). (a) Statistically significant differences (*p* < 0.05) between the control group (C) and extracts. (b) Statistically significant differences (*p* < 0.05) between water formulation (CF) and extracts. (c) Statistically significant differences (*p* < 0.05) between commercial biostimulant (CB) and extracts. 1st term of leaves rosette collection—samples taken for analyses 7 days after the second spraying. 2nd term of leaves rosette collection—samples taken for analyses after harvesting. Abbreviations: UAE, ultrasound assisted extraction; MH, mechanical homogenisation; Hp H, *Hypericum perforatum* L. (St. John’s wort, herb); Sg L, *Solidago gigantea* Ait. (giant goldenrod, leaf); To F, To L, *Taraxacum officinale* (L.) Weber ex F.H. Wigg (common dandelion, flower, leaf); Tp F, *Trifolium pratense* L. (red clover, flower); Ur L, *Urtica dioica* L. (nettle, leaf); Vo R, *Valeriana officinalis* L. (valerian, root).

The highest antioxidant activity, measured using DPPH test, in leaves (Figure 5A) collected in the first term was observed for Hp H UAE (1.6 and 1.5 times more than in C and CB) and the lowest for Vo R UAE (31.8% and 35.3% less than in C and CB), while the highest in leaves harvested in the second term for Sg L MH (21.3% and 62.2% more than in C and CB) and the lowest for Ur L MH (45.2% and 26.7% less than in C and CB). Extract from Tp F UAE increased the antioxidant activity in roots by more than 4 and 2 times in comparison to C and CB, and Ur L MH lowered by 27.8% and 55.2% (Figure 5B).

**Figure 6:**
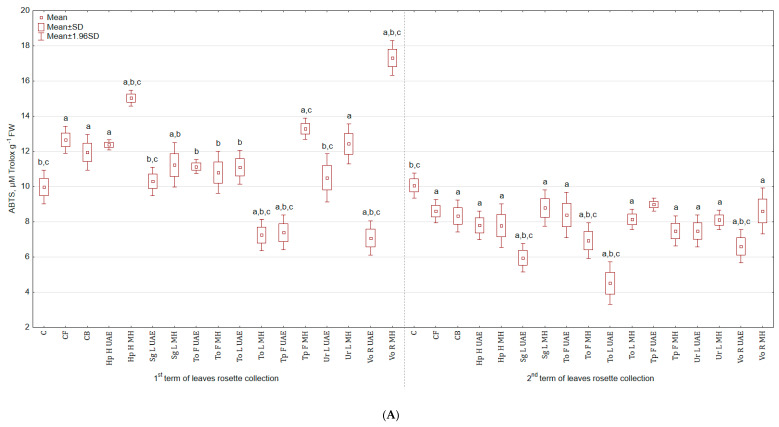

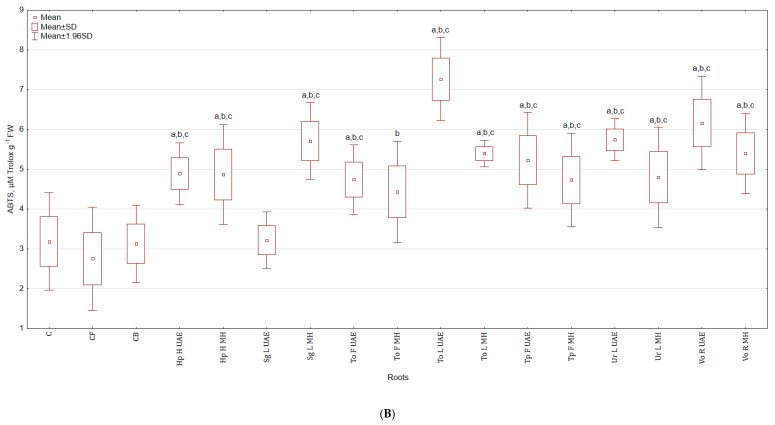
(**A**) Effect of the foliar application of botanical extracts on the antioxidant activity ABTS of celeriac leaves (N = 4). (**B**) Effect of the foliar application of botanical extracts on the antioxidant activity ABTS of celeriac roots (N = 4). (a) Statistically significant differences (*p* < 0.05) between the control group (C) and extracts. (b) Statistically significant differences (*p* < 0.05) between water formulation (CF) and extracts. (c) Statistically significant differences (*p* < 0.05) between commercial biostimulant (CB) and extracts. 1st term of leaves rosette collection—samples taken for analyses 7 days after the second spraying. 2nd term of leaves rosette collection—samples taken for analyses after harvesting. Abbreviations: UAE, ultrasound assisted extraction; MH, mechanical homogenisation; Hp H, *Hypericum perforatum* L. (St. John’s wort, herb); Sg L, *Solidago gigantea* Ait. (giant goldenrod, leaf); To F, To L, *Taraxacum officinale* (L.) Weber ex F.H. Wigg (common dandelion, flower, leaf); Tp F, *Trifolium pratense* L. (red clover, flower); Ur L, *Urtica dioica* L. (nettle, leaf); Vo R, *Valeriana officinalis* L. (valerian, root).

In the case of antioxidant activity evaluated by ABTS test, similar trend as for DPPH test was observed. The highest antioxidant activity in leaves harvested in the 1st term was noted for Vo R MH (73.7% and 44.9% more than in C and CB) and the lowest for Vo R UAE (29.0% and 40.8% less than in C and CB). In the second term of harvest, there were not significant differences between groups sprayed with bio-products and the control group (Figure 6A). However, these differences were observed for roots, e.g., To L UAE increased the antioxidant activity by 127.9% and 132.3% in comparison to C and CB. The lowest value was achieved for Sg L UAE, but it still was higher than for C and CB (Figure 6B).

**Figure 7:**
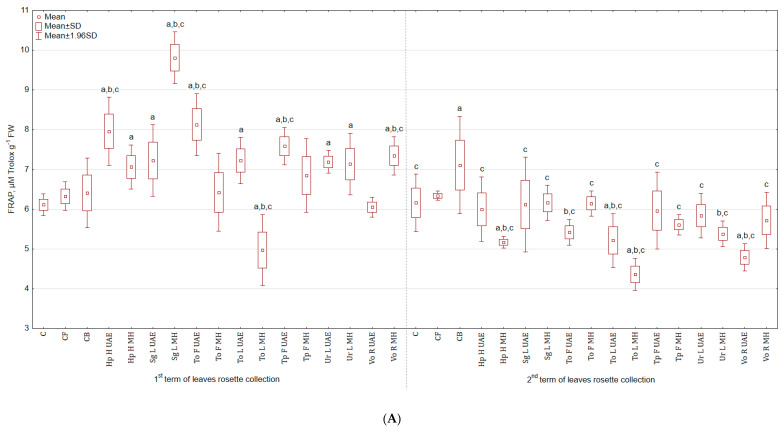

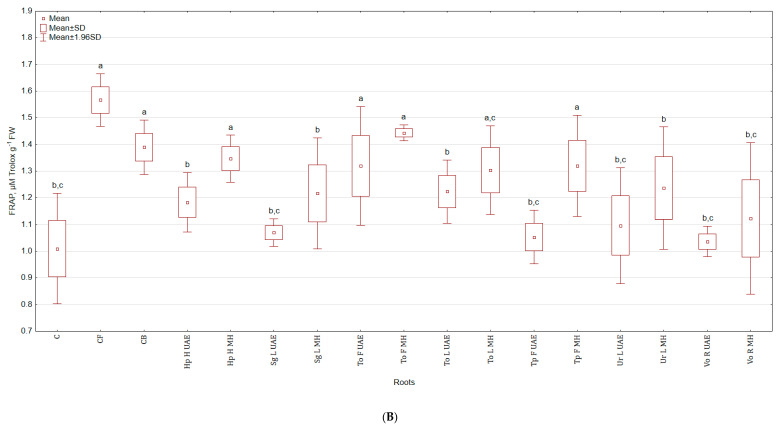
(**A**) Effect of the foliar application of botanical extracts on the antioxidant activity FRAP of celeriac leaves (N = 4). (**B**) Effect of the foliar application of botanical extracts on the antioxidant activity FRAP of celeriac roots (N = 4). (a) Statistically significant differences (*p* < 0.05) between the control group (C) and extracts. (b) Statistically significant differences (*p* < 0.05) between water formulation (CF) and extracts. (c) Statistically significant differences (*p* < 0.05) between commercial biostimulant (CB) and extracts. 1st term of leaves rosette collection—samples taken for analyses 7 days after the second spraying. 2nd term of leaves rosette collection—samples taken for analyses after harvesting. Abbreviations: UAE, ultrasound assisted extraction; MH, mechanical homogenisation; Hp H, *Hypericum perforatum* L. (St. John’s wort, herb); Sg L, *Solidago gigantea* Ait. (giant goldenrod, leaf); To F, To L, *Taraxacum officinale* (L.) Weber ex F.H. Wigg (common dandelion, flower, leaf); Tp F, *Trifolium pratense* L. (red clover, flower); Ur L, *Urtica dioica* L. (nettle, leaf); Vo R, *Valeriana officinalis* L. (valerian, root).

In regard to FRAP test—the antioxidant activity was stimulated in leaves harvested in the first term, but lowered in the second (Figure 7A). The activity was the highest after treatment with Sg L MH for both leaves from the 1st term of harvest (60.6% and 53.0% more than in C and CB), as well as the 2nd term. Extract from To L MH lowered the activity of leaves in both harvesting terms: in the first—by 18.5% and 22.3% and in the second—by 29.3% and 38.7% when compared to C and CB. Roots from experimental groups treated with plant extracts exhibited greater activity than roots from the control group (Figure 7B). For instance, To F MH increased the tested parameter by 42.6% and 3.6% when compared to C and CB.

### 2.5. Nitrates

**Figure 8:**
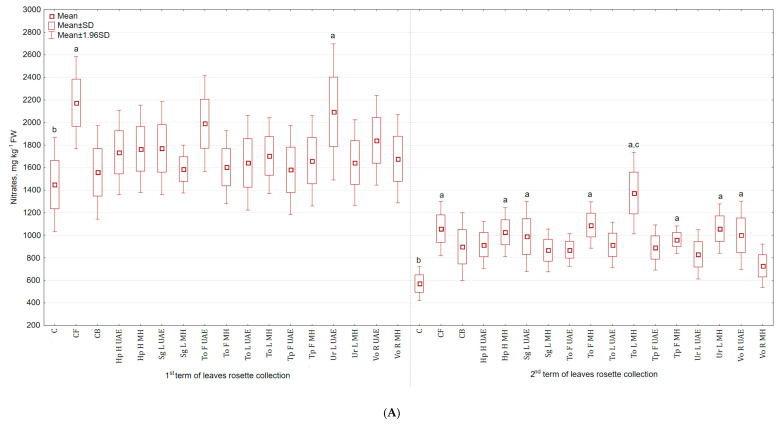

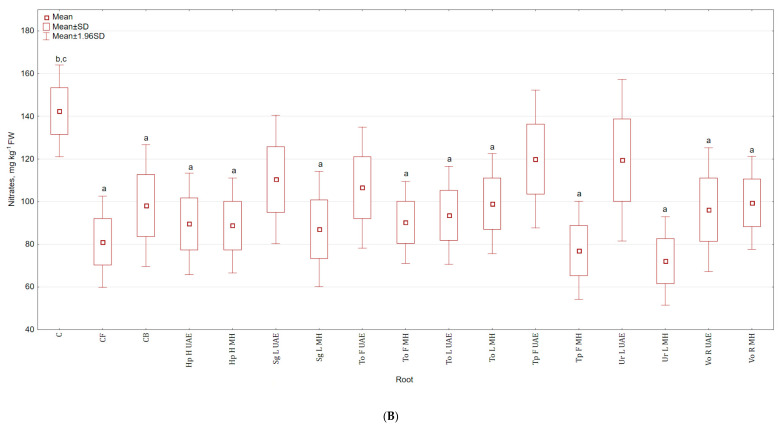
(**A**) Effect of the foliar application of botanical extracts on the nitrates content of celeriac leaves (N = 4). (**B**) Effect of the foliar application of botanical extracts on the nitrates content of celeriac roots (N = 4). (a) Statistically significant differences (*p* < 0.05) between the control group (C) and extracts. (b) Statistically significant differences (*p* < 0.05) between water formulation (CF) and extracts. (c) Statistically significant differences (*p* < 0.05) between commercial biostimulant (CB) and extracts. 1st term of leaves rosette collection—samples taken for analyses 7 days after the second spraying. 2nd term of leaves rosette collection—samples taken for analyses after harvesting. Abbreviations: UAE, ultrasound assisted extraction; MH, mechanical homogenisation; Hp H, *Hypericum perforatum* L. (St. John’s wort, herb); Sg L, *Solidago gigantea* Ait. (giant goldenrod, leaf); To F, To L, *Taraxacum officinale* (L.) Weber ex F.H. Wigg (common dandelion, flower, leaf); Tp F, *Trifolium pratense* L. (red clover, flower); Ur L, *Urtica dioica* L. (nettle, leaf); Vo R, *Valeriana officinalis* L. (valerian, root).

It was found that nitrates content in the above ground parts was higher (in the second term the differences were statistically significant) (Figure 8A), while in roots was lower (Figure 8B) than in the control group. The application of To L MH increased the content of nitrates in leaves (140% and 52.8% more than in C and CB). The lowest content of nitrates was observed in the group treated with Vo R MH (27.5% more than in C and 18.8% less than in CB). The application of Ur L MH decreased the content of nitrates in roots (49.4% and 26.5% less than in C and CB). Spraying celeriac with Tp F UAE caused the highest accumulation of nitrates, but still lower than in the control group (15.8% less than in C and 22.2% more than in CB).

### 2.6. Macroelements, Microelements and Toxic Elements

The examined extracts from St. John’s wort (Hp H), giant goldenrod (Sg L), common dandelion (To F, To L), red clover (Tp F), nettle (Ur L), valerian (Vo R) did not exhibit a significant effect on the increase in the content of macroelements (especially N, P, K, Ca and Mg) in the celeriac leaves when compared with the control group (distilled water) (Table 3). The only exception is the higher sulphur content in leaves in the experimental groups (excluding Ur L MH, Vo R UAE and Vo R MH) than in the control group (C). Generally, almost all examined extracts provided higher content of K, Ca and S in leaves when compared with the commercial biostimulant (CB). Comparing the highest content of elements in the celeriac biomass and the extraction methods, plant extracts obtained by UAE determined a higher content of N, K, Ca and Mg in celeriac leaves, and obtained by MH—P and S. It cannot be clearly indicated which extract promoted to the greatest extent the accumulation of elements in celeriac leaves.

In general, for all examined extracts, the content of macroelements (with the exception of nitrogen) in roots was higher than for the control group (C) and commercial biostimulant (CB) (Table 4). Different extracts were responsible for the highest content of a given element in roots—N (Vo R UAE), P (Tp F MH), K (Sg L UAE), Ca (Ur L UAE), Mg (Sg L UAE) and S (To L MH). It is not possible to clearly state which extraction method will provide a more valuable macroelement composition of roots.

The multielemental analysis of celeriac leaves showed that they were significantly enriched with microelements (Fe, Cu, Zn, Mn and Ni) when compared with the control group (C). The content of Fe in the group treated with To F MH was almost 2.5 times higher than in C, Cu in the group—Vo R UAE by about 10%, Zn in the group—Vo R UAE by 23% higher, Mn in the group—To L MH by 61%, Ni in the groups—Sg L MH and Vo R MH 3 times higher than in the control group (C). For Fe, Cu and Mn, their content in celeriac leaves was generally higher than in the group sprayed with commercial biostimulant (CB).

In the case of celeriac roots, increased manganese content was observed for the all tested extracts in comparison with the control group (C). Moderate impact of botanical extracts on the content of Fe, Zn and Ni in roots was noticed. The weakest effect of the examined extracts on roots composition was in the case of Cu, when compared with the control group (C). The highest content of Fe was in the group treated with To L UAE (1.5 times higher than in C), Cu in the group—To F UAE (by 3.2% higher than in C), Zn in the group—Sg L UAE (by 31% higher than in C), Mn in the group—Tp F UAE (by 33% higher than in C) and Ni in the group—Ur L MH (2 times higher than in C). What is interesting, the content of microelements in roots in the experimental groups was higher, in most cases, than in the group treated with a commercial biostimulant.

Analysing the content of toxic elements (Cd and Pb) in leaves and celeriac roots, their amount was usually lower than in the control group (C), with the exception of Cd content in leaves for all examined extracts (excluding To F UAE and Ur L MH). Higher content of Cd and Pb, both in leaves and roots was observed for Hp H UAE, Sg L UAE, To L UAE and Ur L UAE. But in comparison with the commercial biostimulant, all examined extracts (with a few exceptions) caused higher content of Cd and Pb in the examined samples. On the basis of multielemental composition of leaves and roots of celeriac, extract produced from valerian can be recommended for further research.

### 2.7. Volatile Compounds

It was found that limonene accounted for the largest part of leaves volatile compounds (VCs) and the application of botanical extracts showed a diversified effect on VCs composition (Table 5). Ur L UAE increased the content of this monoterpene by 15.3% and 11.8% in comparison to C and CB, while Tp F UAE decreased it by 18.1% and 20.5%, respectively. β-Myrcene constituted the second major part of oil–in the group treated with Sg L MH, the increment by 57.7% and 31.0%, was observed when compared to C and CB, whereas only Ur L UAE decreased its content by 8.2% and 23.8%, respectively. The content of senkyunolide, the third compound in terms of quantity, was lower in leaves sprayed with bioproducts, contrary to non-treated plants, e.g., Vo R UAE (21.9% less than in C but 88.2% more than in CB) and Sg L MH (84.7% and 63.1% less than in C and CB). A similar pattern was observed for (*Z*)-β-ocimene –Hp H MH decreased its content by 19.2% in comparison to C but increased by 30.4% when compared to CB. The amount of 2-undecanone was stimulated by Hp H MH (12.3% more than in C, 5.3% more than in CB), Tp F UAE (10.8% more than in C, 4.0% more than in CB), Sg L MH (5.8% more than in C, 0.8% less than in CB), and Vo R UAE (5.5% more than in C, 1.0% less than in CB). Tp F UAE increased the content of 3-butylphthalide (21.0% and 51.2% more than in C and CB, respectively) and neocnidilide (163.8% and 160.2% more than in C and CB). While, Sg L MH lowered the quantity of 3-butylphthalide (42.1% less than in C, 27.7% less than in CB). The decrease in neocnidilide amount was observed in the group treated with Sg L MH (37.7% less than in C, 38.6% less than in CB) and To L UAE (39.4% less than in C, 40.2% less than in CB). Overall, it can be stated that the mixture of compounds extracted from higher plants modified the volatile compound composition of celeriac leaves.

### 2.8. Fatty Acids

The composition of fatty acids (FAs) in celeriac roots is presented in Table 6. It can be seen that bioproducts exert diverse influence on the FAs constitution, of which the largest part accounted for 9, 12-hexadecadienoic acid (methyl ester) and hexadecanoic acid (methyl ester). Most of the botanical extracts decreased the content of pentadecanoic acid (methyl ester), hexadecanoic acid (methyl ester; 15-methy-, methyl ester; 14-methyl, methyl ester), heptadecanoic acid (methyl ester), octadecanoic acid (methyl ester), 11-octadecenoic acid (methyl ester), methyl 18-methylnonadecanoate, docosanoic acid (methyl ester), tricosanoic acid (methyl ester), and tetracosanoic acid (methyl ester; ethyl ester). The content of 9, 12-hexadecadienoic acid, was stimulated by all of the tested plant extracts in comparison to C, for example: the highest amount was achieved in groups treated with CB, Hp H MH, Tp F MH, To F MH (more by 24.0%, 21.6%, 21.6%, 21.2%, respectively) and the lowest with To L MH (more by 13.0%). On the other hand, the content of hexadecanoic acid was lowered after all foliar sprays, for instance for CB by 9.2%, and for extracts from 9.4% (To F UAE) to 35.7% (Vo R UAE). Sg L UAE increased the content of tetradecanoic acid (ethyl ester), tetradecanoic acid (12-methyl-, methyl ester), pentadecanoic acid (14-methyl-, methyl ester), *Z*-9-hexadecenoic acid (methyl ester) by 41.8%, 57.3%, 30.2%, and 24.1% more than in C, respectively, and more by 111.1%, 126.1%, but less by 18.5% than in CB, respectively. Vo R UAE enhanced the content of dodecanoic acid (methyl ester), *Z*-9-hexadecenoic acid (methyl ester), and *Z, Z, Z*-9, 12, 15-octadecatrienoic acid (methyl ester) by 123.3%, 186.5%, 57.5% in comparison to C, and by 81.1%, 88.0%, 30.1% to CB. This product decreased the content of pentadecanoic acid (14-methyl-, methyl ester) by 18.5% than in C (but more by 31.6% than in CB) and hexadecanoic acid (methyl ester) by 35.7% and 29, 2% than in C and CB, respectively. To L MH increased the content of 9*Z*-9-octadecenoic acid (ethyl ester) by 43.5% and 78.4% when compared with C and CB, respectively.

## 3. Discussion

A wide range of conducted studies have dealt with the foliar application of botanical extracts and their impact on the yield and quality traits of both leaves and roots of celeriac. Our novel results proved that this type of products can exhibit positive effects on crop plants. The crop yield is determined by many factors, e.g., husbandry (e.g., planting method, population density, time of sowing, fertilisation, irrigation, weed control, cultivar, previous crop), stress (e.g., drought, salinity, pests and diseases), intercropping, genetic [22,23], climatic (e.g., temperature, precipitation, humidity, solar radiation, atmospheric gases, wind) [24,25] and soil (e.g., moisture, aeration, temperature, mineral and organic matter, organisms, pH) [26]. The weather during the field experiments was characterised by high temperatures and low rainfall which were not optimal conditions for the growth of celeriac–what can be observed in the obtained yield (despite of profuse watering). On the other hand, these stress conditions allowed to evaluate the impact of botanical extracts. The suitability of the use of biostimulants in the cultivation of crops was emphasised by many researchers worldwide [7,27,28] and it was shown that plant growth biostimulants are particularly effective if stress conditions prevail.

Nowadays, the interest in the use of ecological products in modern horticulture is constantly growing. The up-to-date published literature concerning the use of natural raw materials for the production of bio-products is summarised in Table 7. Furthermore, an interesting aspect of this research is the possibility to compare the results obtained in the field trials with previously published findings, that concerned germination tests performed under laboratory conditions [14,15]. In these tests, the effect of examined extracts (obtained through ultrasound assisted extraction) on the growth and chemical composition of white head cabbage seedlings was evaluated. In the laboratory experiments, the impact of different concentrations of botanical extracts (0.1, 0.5, 1.0, 2.5%) was examined, but for this comparison we will focus only on the concentration tested in the field trials – 0.5%. All these studies proved the beneficial effects of botanical extracts on crop plants.

The conducted field trials showed that the application of To F UAE (for root diameter < 5 cm), To L MH (for root diameter 5–9 cm) and Hp H MH (for root diameter 9–13 cm) promoted the fresh weight of leaves rosette. On the other hand, in laboratory experiments, among selected botanical sources (St. John’s wort, giant goldenrod, common dandelion, red clover, nettle, valerian), the highest fresh weights of shoots were achieved for Ur L and To F. For the treatment with extract from Hp H, the differences were not statistically significant. The heaviest roots obtained in field trials were reported for Tp F UAE (for root diameter < 5 cm), To L MH (for root diameter 5–9 cm) and Hp H MH (for root diameter 9–13 cm) whilst in germination test for all of the evaluated extracts (e.g., Vo R, Sg L, To F, To L). In case of the dry weight of shoots of celeriac grown in field trials—bio-product based on Tp F (MH) showed the greatest stimulating activity, while for roots—all used extracts increased the dry weight of roots (especially: To F MH, To L MH, Tp UAE, Vo R MH). In laboratory conditions, the highest DW of shoots of white head cabbage seedlings was observed in groups sprayed with To F and Ur L (there were no significant differences after application of Hp H), whereas for roots by all the botanical extracts (e.g., Vo R). The higher weight was also noted after application of extracts based for example on: moringa leaves, red grape skin, blueberry fruits, hawthorn leaves, bee-honey, garlic cloves, olive leaves, pomegranate leaves, common guava leaves, liquorice root, borage leaves and flowers, cultivated tobacco leaves, apple seeds, colza seeds, rice husks (Table 6). Enhanced efficiency of N uptake, reduced chlorophyll degradation and leaf ageing may lead to a higher greenness index of celeriac leaves [29,30,31,32]. The highest content of chlorophyll *a* + *b* can be observed after the second spraying with examined botanical bio-products, but simultaneously no significant impact on the SPAD values can be noted. It looks conversely in the case of plants sprayed three times with preparations—lower content of chlorophyll *a* + *b* but significantly higher for SPAD values. The efficient photosynthesis is extremely important, because it determines the crop yield and the effectiveness of capturing the light and transforming it into the biomass [33]. The chlorophyll in leaves is a major indicator of the leaf greenness, and is frequently determined to verify the nutrient deficiencies (e.g., nitrogen and the changes in its content) [34]. There is a substantial link between the amount of chlorophyll and leaf nitrogen. The content of this pigment and leaf dry weight are increased by appropriate fertilisation, in particular with nitrogen-containing compounds [35,36,37]. In field tests, the highest content of pigments was observed after application of To F UAE. The greenest leaves were observed in the group treated with To L MH. Most of the examined extracts enhanced the content of chlorophyll *a* + *b* in white head cabbage seedlings (e.g., To L and Hp H). Similar trends were observed for measurements of the greenness index of leaves. The highest content of carotenoids was in groups treated with Hp H, Tp F and Vo R. The increment in pigments content was also observed after usage of extracts from e.g., moringa leaves, red grape skin, blueberry fruits, hawthorn leaves, bee-honey, garlic cloves, sugar beet, lantana, liquorice root, palm pollen grains, borage leaves and flowers, and also carrot roots (Table 7).

Plant growth biostimulants, in addition to affecting the content of nitrates in the plant, also affect the content of vitamins, e.g., vitamin C. An increased level of nitrates in soil tends to decrease the vitamin C amount in plants [38]. Therefore, the adequate potassium levels are required to maintain the right amount of this vitamin [39]. The physiological functions of this macroelement remain not entirely understood but it is well known that it is required for proper plant growth, metabolic processes in a cell, protein biosynthesis, assimilates transportation, osmoregulation of cells and in stomata movement. It has an important role in transportation of NO_3_^−^ when there is insufficient amount of K, plants amassed nitrogen compounds along with toxic amines [40]. Other factors that may affect its content in plants include genotype, weather, cultivation and harvesting methods, maturity as well as postharvest treatments. The high light intensity and less frequent irrigation positively affect the content of vitamin C [38,41]. The conducted research showed that the application of botanical extracts increased the content of vitamin C in celeriac leaves – mostly after the second application (e.g., Vo R UAE and Hp H MH). In the case of roots, in majority of cases the differences were not statistically significant. The highest amount of this vitamin was observed in the group treated with Ur L UAE. The literature indicates that bio-products based on e.g., moringa leaves, palm pollen grains, apple seeds, colza seeds, rice husks can also increase the content of ascorbic acid (Table 7).

High nitrogen fertilisation may also lead to the lower phenolic content in cultivated plants [42]. In our research, this tendency was noted for celeriac leaves while in roots the higher content of polyphenols may be noted. In plants, these compounds influence their growth and development (e.g., seed germination, cell division, synthesis of photosynthetic pigments), participate in signal transduction from roots to shoots and nutrient mobilization (Ca, Mg, K, Zn, Fe, Mn), improve nutrient uptake through chelation of metallic ions, enhance active absorption sites, and soil porosity, increase tolerance to environmental stresses (e.g., drought, salinity, temperature, pesticide, UV radiation). Due to the antioxidative properties and capability of scavenging free radicals they reduce cell membrane peroxidation, and protect cells from of oxidative stress [43]. The root of celeriac contains about 40–90 mg per 100 g of fresh weight of polyphenols expressed as gallic acid equivalent [44,45,46]. Most of the tested extracts did not increase the content of TPC in leaves but increased in roots. A majority of the tested botanical extracts decreased the content of total phenolic compounds in cabbage seedlings. However, the application of extract based on nettle increased their content in comparison to control groups. Similar trend was observed for celeriac leaves grown in the field. The highest content of TPC was in groups treated with Tp F MH and Ur L MH. In the case of celeriac roots—most of extracts significantly increased the content of polyphenols (e.g., Hp H UAE). It was shown that, extracts obtained from e.g., moringa leaves, red grape skin, blueberry fruits, hawthorn leaves, borage leaves and flowers, alfalfa plant exhibited diverse stimulation activity of phenolic compounds in cultivated plants (Table 7).

A similar trend as for phenolic compounds was observed in the case of antioxidant properties. Botanical extracts did not increase the antioxidant activity of cultivated vegetables measured using DPPH test in the laboratory experiments but increased in field tests. The increment was noted for shoots harvested in the second term but only after treatment with Sg L MH. However, most of the extracts increased the antioxidant activity in roots, where the best results were achieved for Tp F UAE. In germination tests, majority of examined extracts increased the antioxidant activity measured using ABTS test, especially To F and Ur L. The significant changes were observed only in celeriac roots (e.g., To L UAE). In FRAP test on cabbage seedlings, only Ur L increased the activity. Similar trend can be observed in field test—only To F MH increased the activity in celeriac roots. As reported by other authors, bio-products based on e.g., moringa leaves can increase the radical scavenging activity, activities of antioxidant enzymes, antioxidant activity contents; red grape skin, blueberry fruits, hawthorn leaves, borage leaves and flowers, alfalfa plant—the phenylalanine ammonia lyase activity; olive leaves, pomegranate leaves, common guava leaves—the protease and catalase activities; sugar beet—the antioxidants’ activities; liquorice roots—the activities of catalase, peroxidase, ascorbate peroxidase, superoxide dismutase and glutathione reductase; palm pollen grains—the antioxidant enzyme activities; apple seeds, colza seeds, rice husks—the antioxidant capacity (Table 7).

The highest levels of nitrates are found in green leafy vegetables (much greater than root and fruit vegetables) [47]. As an example, celeriac contains high concentrations of these compounds (1000–2500 mg·kg^−1^) [47]. In our research, the increased nitrates content in the cultivated celeriac has been also observed. It was found that their content in leaves rosette from the second term of collection was significantly higher (the highest for e.g., To L MH), while in roots was lower than in the control group (the lowest for e.g., Vo R MH). On the other hand, the extracts based on borage leaves and flowers did not exert significant impact on nitrate levels (Table 7).

In the case of macro- and microelements, both celeriac leaves and roots were enriched with these elements – for example, after the application of extracts based on Vo R. This is highly important in view of the fact that both parts of celeriac are edible and can be a source of well bioavailable minerals for humans. Biostimulants of plant growth are known to enhance the nutritional profile of plant biostimulant-treated plants through increasing the availability of soil nutrients, plant nutrients uptake and their assimilation and translocation [48,49]. The increased content of elements (N, P, K) in plants was also noted after treatment with extracts produced from e.g., moringa leaves and liquorice root (Table 7).

Essential oils are powerful compounds from natural sources, ordinarily plants, which are valued for their healing properties and prevention and treatment of cancer and cardiovascular diseases as well as antioxidant, antiviral, antidiabetic, and antibacterial activities [50,51,52,53]. It has been also shown that the application of biostimulants has a significant impact on the oil amount in plants [54]. Our research proved that botanical extracts induced diversified effect on leaves volatile compounds composition. In Table 7 it was shown that moringa leaves extracts increased the volatile oil yield and oil components. In the case of palm pollen grains, increase in the content of essential oils was observed

The ω−3 fatty acids are especially important because they play a major role in the prevention and treatment of coronary artery disease, hypertension, diabetes, arthritis, other inflammatory and autoimmune disorders, and cancer [55]. The literature shows that celeriac contains 0.079 g of the total saturated fatty acids, 0.058 g of total monounsaturated fatty acids, and 0.148 g of total polyunsaturated fatty acids [56] and celery contains 15% of fatty oil, with fatty acids: petroselenic (64.3%), oleic (8.1%), linoleic (18%), linolenic (0.6%), and palmitic acids [57]. It can be seen that botanical extract exerted diverse influence on the FAs composition. The largest part was accounted for 9, 12-hexadecadienoic acid (methyl ester) and hexadecanoic acid (methyl ester). The content of the first acid was stimulated by all of the tested plant extracts (e.g., Hp H MH, Tp F MH, To F MH) while the content of the second acid was lowered by all foliar sprays (e.g., Vo R UAE).

It can be noticed that depending on the type of tests (in controlled or real environment) and on the plant species, the examined bio-products influenced their growth and chemical composition differently. It shows how extremely important is the thorough examination of new botanical products before launching them on the market. The detailed product safety data sheets should be prepared for their optimal use. It can be concluded that biostimulants are a promising future in the functional plant nutrition linked to the increased quantity and quality of yield, free from pesticide residues and rich in healthy substances.

## 4. Materials and Methods

### 4.1. The Tested Raw Materials and Botanical Extracts Production

The raw materials used for the production of botanical extracts were selected based on our previous studies [14,15]. Seven sources (candidates) of bioactive compounds were chosen: St. John’s wort (*Hypericum perforatum* L.) (herb) (marked as: Hp H), giant goldenrod (*Solidago gigantea* Ait.) (leaf) (Sg L), common dandelion (*Taraxacum officinale* L. (L.) Weber ex F.H. Wigg) (flower, leaf) (To F, To L), red clover (*Trifolium pratense* L.) (flower) (Tp F), nettle (*Urtica dioica* L.) (leaf) (Ur L), valerian (*Valeriana officinalis* L.) (root) (Vo R). For the production of bio-products, two methods were applied: ultrasound assisted extraction (UAE) (using homogeniser UP 50 H, Hielscher Ultrasonics GmbH, Teltow, Germany) and mechanical shearing combined with sonic energy (MH) (using Unidrive X1000 Homogenizer Drive, Ingenieurbüro CAT, Ballrechten-Dottingen, Germany). The ratio of dried and ground (500 μm mesh size) biomass to deionised water was 1:20 (*w*/*v*). For the UAE method, the well stirred mixture of a given biomass and water was soaked for 30 min at room temperature, and afterwards sonicated (30 min), whereas for the MH method, the mixture was homogenised (mechanical shearing and sonic energy) for 1 min (28000 rpm) and then centrifuged (4500 rpm, 10 min, Heraeus Megafuge 40, rotor TX-750, Thermo Scientific, Waltham, MA, USA). The obtained supernatants constituted 100% liquid extracts that were prepared freshly before using in field trials or were stored in dark glass bottles in a refrigerator. In order to obtain a safe product that will not deteriorate over a period of time and will deliver more bioavailable compounds the proper formulations were prepared. The final product consisted of: active ingredient (extract, 0.5% *w*/*v*), adjuvant (Protector, 0.02% *w*/*v*), antioxidant agent (L-ascorbic acid, 0.15% *w*/*v*), preservative (potassium sorbate, 0.1% *w*/*v*) and water (up to 100%). These formulations are aimed at increasing the droplet adhesion on leaves’ surface, enhancing the transport and absorption of nutrients in the plant by elongating the time of wetting the leaf surface.

### 4.2. The Field Trials

The field trials were conducted at The Research and Teaching Station of Vegetable and Ornamental Plants in Psary (51°11′25.27′’ N 17°2′3.08′’ E) belonging to Department of Horticulture at Wrocław University of Environmental and Life Sciences. The weather conditions are presented in Figure S1. The impact of botanical extracts was assessed on celeriac (*Apium graveolens* L. var. *rapaceum*). It was chosen as a model plant because of the increasing production due to its high content of healthy components and taste attributes. The experiments were performed in randomised complete blocks in three replications for each tested product. The fine clay soil (pH 7.36, EC 153.4 μS·cm^−1^), containing 1.8% humus, 24.5 mg P, 118.7 mg K, 436.7 mg Ca and 36 mg Mg in 1 dm^3^, was pre-plant fertilised with Hydrocomplex Yara Mila (250 kg·ha^−1^) and ammonium saltpetre (330 kg·ha^−1^), and top dressed with ammonium saltpetre (two times, 82.5 kg·ha^−1^ each). During plant growth, the typical cultivation treatments (e.g., regular mechanical weeding, irrigation) were made. Additionally, herbicides (Fusilade Forte 150 EC, Dispersive Afalon 450 SC), and fungicides (Scorpion 325 SC, Tattoo C 750 SC) were applied once. The seeds of celeriac (cultivar ‘Neon’, Semo) were sown in March 26 in a greenhouse, seedlings were pricked out in April 17, planted to the soil in spacing 45 cm × 20 cm (plot size: 2.88 m^2^; 32 plants per plot) in June 5, and harvested in October 9, 2018. The plant extracts were applied at a dose of 600 L·ha^−1^ in: July 15, July 22, and August 1, 2018. The samples were taken for analysis 7 days after the second spraying (1st term of leaves rosette collection) and after harvesting (2nd term of leaves rosette and roots collection). During the application of botanical extracts, the circadian rhythm of plants was taken into consideration. The treble foliar sprays were performed on sunny, windless days in the morning when the stomata were open and the assimilation rate was at its peak. Plants sprayed with water (C), formulation with water (CF) and commercial biostimulant (CB) were taken as control groups.

### 4.3. Chemicals

Acetone, calcium carbonate, sodium carbonate, ethanol, potassium persulphate, and sodium acetate were purchased from IDALIA (Radom, Poland), Folin-Ciocalteu’s phenol reagent, Trolox, gallic acid, diphenyl-2-picrylhydrazyl (DPPH), azino-bis-3-ethyl-benzthiazoline-6-sulphonic acid (ABTS), ferric reducing antioxidant power (FRAP), and tripyridyl-S-triazine (TPTZ) from Archem (Łany, Poland), acetic acid, activated carbon, oxalic acid, ascorbic acid, sodium bicarbonate, 65% nitric acid, ammonium molybdate tetrahydrate, ammonium metavanadate, magnesium nitrate, barium chloride dihydrate, cyclohexane, sodium sulphate from CHEMPUR (Piekary Śląskie, Poland), 2, 6-2, 6-dichlorophenolindophenol sodium salt hydrate from Acros Organics (Argenta; Poznań, Poland), hydrochloric acid (38%), chloroform and methanol from Stanlab (Lublin, Poland), standard solutions and detergent Tween TM 80 from Merck (Darmstadt, Germany), 2-undecanone and BF_3_/MeOH from Sigma-Aldrich (Saint Louis, MO, USA), hexane and sodium bicarbonate from UQF (Wrocław, Poland), n-hexane (99%) from POCH Basic (Gliwice, Poland), helium from Air Products (Warsaw, Poland), potassium hydroxide from Avantor (Gliwice, Poland). The reagents were of analytical grade.

### 4.4. The Photosynthetic Pigments, Greenness Index of Leaves, and Leaves Colour

For the determination of the content of chlorophyll *a* + *b* (mg·100 g^−1^ fresh weight, FW) and carotenoids (µg·100 g^−1^ FW) [14,15,83], the freshly collected celeriac leaves (0.4 g) were comminuted to a smooth paste in a mortar with a few drops of acetone (80%), pinch of sand and calcium carbonate. Then, the mixture was filtered using Schott filter and vacuum pump, quantitatively transferred to a volumetric flask (50 mL) and filled up with the solvent. The measurements (in four replicates) of absorbance (663, 645, and 470 nm) were made immediately after preparing the solutions with the use of portable visible spectrophotometer (HACH DR1900, Berlin, Germany). The formulas for the calculations are presented in our previous study [14,15].

The survey of the greenness index (in 10 replicates) of leaf blades was performed using SPAD 502 Plus Chlorophyll Meter (Konica Minolta, Osaka, Japan).

The colour of leaves (in 10 replicates) was assessed using MiniScan (Hunter Lab EZ, Reston, VA, USA). The *L* value for each scale indicates the level of light (numbers from 51 to 100) or dark (numbers from 0 to 50), the *a* value indicates redness (positive number) or greenness (negative number), and the *b* value yellowness (positive number) or blueness (negative number). All these three values are required to completely describe leaves’ colour.

### 4.5. Vitamin C

The content of vitamin C (mg·100 g^−1^ FW) was determined according to the modified procedure described by [84,85,86]. The fresh celeriac leaves (~5 g) and roots (~15 g) were homogenised (Koenic blender) with oxalic acid (200 mL, 2%). The obtained solutions were filtrated and filtrates (10 mL) were collected and titrated (in 4 replicates) with a solution of 2,6-dichlorophenolindophenol (Tillmans’ reagent) till the light pinkish colour occurred and lasted for at least 30 s.

### 4.6. Total Phenolic Compounds

The total phenolic compounds (TPC) content (mg of gallic acid equivalents (GAE)·100 g^−1^ FW) was determined in accordance to the Folin-Ciocalteu method proposed by Jałoszyński et al. [87] with slight alterations [14,15]. The fresh and fragmented (using Thermomix) shoots and roots (~2 g) were placed in 50 mL falcon tubes and the aqueous methanol (20 mL, 80%) was added. The test tubes were sonicated (Bandelin Sonorex RK 100 H, Berlin, Germany) for 15 min and centrifuged (10 min, 4500 rpm) (Heraeus Megafuge 40, rotor TX-750, Thermo Scientific). To the supernatants (0.1 mL), the Folin-Ciocalteu’s phenol reagent (0.2 mL) and distilled water (2 mL) were added and left to stand at room temperature in the dark for 3 min. Afterwards, sodium carbonate (1 mL, 20%) was added, and the reaction mixtures were kept in the dark for 1 h. The absorbance at 765 nm was measured (HACH DR1900 spectrophotometer, four replicates).

### 4.7. The Antioxidant Activity (DPPH, ABTS, and FRAP)

For the determination of antioxidant activity, ten-fold diluted supernatants, obtained for the analyses of TPC content, were used.

The DPPH radical scavenging activity (µM Trolox 1 g^−1^ FW) was conducted as described by Yen and Chen [88] with minor changes [14,15]. The radical stock solution of DPPH was freshly prepared by dissolving in ethanol. Each supernatant (0.5 mL) was mixed with ethanol (1.5 mL) and DPPH solution (0.5 mL), and then stirred and incubated at room temperature in the dark. Absorbance at 517 nm was determined after 10 min (in 4 replicates).

The ABTS assay (µM Trolox · g^−1^ FW) was determined following the modified method of Re et al. [89] and Almeida et al. [90] as described by Godlewska et al. [14] and Godlewska et al. [15]. The ABTS radical cation was made by the reaction of aqueous ABTS solution (5.0 mL, 7 mM) with potassium persulfate solution (88 μL, 140 mM). Before being used, the mixture was kept in darkness at 29 °C for more than 14 h. The blue-green ABTS solution (3.0 mL) (diluted in ethanol to obtain the absorbance of 0.7 *±* 0.02 units at 734 nm) was added to supernatants (30 μL) and left for 6 min without access to light. The measurements were made in 4 replicates.

The FRAP antioxidant capacity (µM Trolox · g^−1^ FW) was examined according to the method presented by Benzie and Strain [91] with small modifications by Godlewska et al. [14,15]. For the preparation of the ferric reducing antioxidant power (FRAP) reagent, the acetate buffer (300 mM), TPTZ (10 mL in 40 mM HCl) and FeCl_3_·6H_2_O (20 mM) in a ratio of 10:1:1 at 37 °C were mixed. The supernatants (1 mL) were mixed with FRAP reagent (3.0 mL). After 10 min, the absorbance (593 nm) was measured (in four replicates).

### 4.8. Nitrates

The nitrates content (mg·kg^−1^ FW) was determined according to methodology described by Krężel and Kołota and by Nowosielski [85,92]. The dried (50 °C) and ground (500 µm) samples (0.4 g) were mixed with acetic acid (100 mL, 2%) and activated carbon (0.5–1.0 g) and put on an laboratory shaker (Thermo Scientific MAXQ 2000, Dubuque, IA, USA) and were shaken for 30 min (150 rpm). Then, the solution was filtrated (the first drops were not collected) and the content of nitrates was measured in 4 replicates using ionometer (Thermo 5 Star Orion, Beverly, MA, USA) with an ion-selective electrode.

### 4.9. Macroelements, Microelements and Toxic Elements

The dried and ground biomasses were used to assess the content of macroelements (P, K, Ca, Mg) (mg·kg^−1^ DW), microelements (Mn, Fe, Cu, Zn, Ni) (mg·kg^−1^ DW), and heavy metals (Cd, Pb) (mg·kg^−1^ DW). Samples were mineralised in an oven at 450 °C for 8 h (CZYLOK, Jastrzębie-Zdrój, Poland). The obtained ash was digested in 65% HNO_3_ and evaporated on a heating plate at 110 °C for 6 h. The contents of the evaporating dish were then dissolved in 1 M HNO_3_ and transferred quantitatively to the flask. The content of P was determined by colorimetric method (400 nm), resulting in the yellow coloured complex with molybdate and ammonium metavanadate (Cecil CE 2011 photometer). The content of K, Ca, Mg, Mn, Fe, Cu, Zn, Cd, Pb and Ni was analysed by atomic absorption spectrophotometry (ASA) (Varian Spectra AA 220/FS instrument, Mulgrave, Australia) maintaining the parameters specific to individual elements.

The sulphur content was determined by the method of Butters and Chenery [93] with the modification of Bielecki and Kulczycki [94]. The essence of this method is the oxidation of sulphur, which occurs in organic compounds and its determination on the basis of the turbidity of the solution of sulphate content precipitating as barium sulphate. In the modified method, the sulphur mineralisation stage was left unchanged. However, the stage of preparation of the analysed solution for measurements has been modified. Instead of the barium chloride crystals BaCl_2_ 2H_2_O, barium reagent was used, which is a solution of barium chloride and Tween TM 80 detergent. Quantitative measurements were made on a Cecil CE 2011 photometer (Cambridge, UK) at 400 nm. The results obtained in the modified method are about 50% higher than those obtained using the Butters and Chenery method and similar to those obtained using the LECO analyser.

The nitrogen content was evaluated according to the Kjeldahl method described by Jones [95]. The samples were mineralised in concentrated sulphuric acid with the addition of selenium (as a catalyst) and hydrogen peroxide (as an oxidising agent). After mineralisation, the nitrogen in samples was in a form of acid ammonium sulphate. The cooled solution was mixed with a strong base solution and the emitted ammonia was distilled into a saturated boric acids solution with the addition of a mixed indicator (methyl red and bromocresol green). The solution of ammonia in a boric acid was determined by titration with a standard hydrochloric acid solution (0.01 M) until an initial colour was obtained (such as before ammonia absorption).

### 4.10. Volatile Compounds

The volatile compounds analyses were made in accordance to modified procedure described by Calín-Sánchez et al. [96]. Frozen celeriac leaves (~15 g) and distilled water (100 mL) were transferred to a 250 mL round-bottom flask and boiled in a heating mantle. At the beginning of the hydrodistillation process, 1 mL of cyclohexane containing 1 mg of 2-undecanone, as internal standard, was added to retain the volatile compounds distilled from the samples. The distillation process was performed using the Deryng apparatus. After 50 min of extraction, the solvent containing the volatiles was transferred into 2.5 mL vials and stored in −18 °C until the chromatographic analyses were conducted (in three replicates).

The volatile compounds were isolated and identified using a gas chromatography coupled with mass spectrometry (GC-MS, GCMS QP 2020, Shimadzu, Kyoto, Japan) with a capillary column Zebron ZB-5 (30 m, 0.25 mm, 0.25 μm; Phenomenex, Torrance, CA, USA). The scanning was performed from *m/z* 35 to 320 in 70 eV electronic impact at 3 scans s^−1^. Helium, at a flow rate of 1.11 mL min^−1^ was used as a carrier gas. The selected split ratio was 1:20 and the over program was: (a) 45 °C as initial temperature; (b) rate of 2 °C min^−1^ to 150 °C; (c) rate of 15 °C min^−1^ to 270 °C for 5 min. The temperatures of injector and interface were 260 °C and 250 °C respectively. The injection volume was 1 μL. The identification of most of the compounds were based on three different methods: (a) the retention times of unknown compounds with authentic standards; (b) the retention indices (RI) of compounds to be identified; (c) mass spectra, with indices of similarity above 90% (FFNSC and NIST17 spectral libraries collection and authentic chemicals).

### 4.11. Fatty Acids

The lipid fraction of dried celeriac roots was obtained according to the procedure described by Folch et al., Maslak et al., Gholami Zali et al. [97,98,99]. For the preparation of the lipid fraction, the dried and ground celeriac roots (100 mg) were macerated with chloroform (5 mL). Then, the obtained extracts were filtered and evaporated in a vacuum. The extracted non-polar lipid fraction (25 mg) was saponified (5 min at 65 °C) with 0.5 M KOH/MeOH solution (2 mL) and subjected to methylation (10 min at 65 °C) by adding 14% (*v*/*v*) BF_3_/MeOH (2 mL). Next, the distilled water (5 mL) was added and the methyl esters of fatty acids were extracted with hexane (10 mL). The mixture was washed with 10% sodium bicarbonate (10 mL) and dried over anhydrous sodium sulphate. The organic phase was evaporated under reduced pressure, and dissolved in hexane (200 µL), transferred to vials and stored in -27 °C until chromatographic analyses. The profile of fatty acid methyl esters was analysed by gas chromatograph coupled with a mass spectrometer (Shimadzu GCMS QP 2020). The separation was made using capillary column Zebron ZB-FAME (60 m, 0.20 mm, 0.20 μm; Phenomenex). The GC-MS analyses were carried out according to the following parameters: scanning was performed in the range from *m/z* 40 to 400 in electron impact (EI) at 70 eV, in the mode of 3 scans s^−1^. Analyses were conducted using helium as carrier gas at a flow rate of 1.8 mL min^−1^ in a split ratio of 1:10 and the following program: a) 80 °C for 2 min; b) rate of 3.0 °C min^−1^ from 80 to 180 °C; c) rate of 8 °C min^−1^ from 180 to 240 °C. The injector was kept at 280 °C. The identification of compounds were based on 2 an independent methods: (i) retention times with authentic chemicals (Supelco, 37 Component FAME mix); (ii) obtained mass spectra, with available library (Wiley NIST17, similarity index > 90%).

### 4.12. Statistical Analyses

The statistical analyses of results were conducted using the STATISTICA program ver. 13.3 (TIBCO Software Inc., Tulsa, OK, USA). The Shapiro-Wilk test was used to verify the normality of the data. In the case of normal distribution, the Brown-Forsythe test was applied to evaluate the homogeneity of variance, and the differences were assessed with the Tukey’s Honest Significant Difference (HSD) test (for p lower than 0.05 the data were significantly different). The Kruskal–Wallis test was used for data not normally distributed. Statistically significant differences between botanical extracts and water (C) were marked with “a”, between formulation (CF) with “b” and between commercial biostimulant (CB) with “c”.

## 5. Conclusions

The research carried out represents a valuable source of data which allowed to eliminate the gap between laboratory data on single plant extracts (presented in papers by Godlewska et al. [41] and Godlewska et al. [42]) and field tests combined with fertilisers. This study provides a crucial knowledge about the effects of botanical extracts on crops under real-field conditions. The biostimulating properties of seven raw materials: St. John’s wort (*Hypericum perforatum* L.) (herb), giant goldenrod (*Solidago gigantea* Ait.) (leaf), common dandelion (*Taraxacum officinale* (L.) Weber ex F.H. Wigg) (flower, leaf), red clover (*Trifolium pratense* L.) (flower), nettle (*Urtica dioica* L.) (leaf), valerian (*Valeriana officinalis* L.) (root) were examined. Extracts were produced using two methods: ultrasound assisted extraction (UAE) and mechanical shearing combined with sonic energy (MH). Plants sprayed with water (C), water formulation (CF) and commercial biostimulant (CB) constituted control groups.

The presented multidisciplinary approach (from raw materials, through extracts production, to the final formulations and their application on crop plants characterised in terms of chemical composition) demonstrated that botanical extracts based on plants commonly occurring in Europe exerted diverse biostimulating effects on celeriac growth and physiological parameters. They can be used to achieve higher yield (e.g., Hp H MH), dry weight (e.g., Tp F UAE, Tp F MH), antioxidant activity (DPPH assay: e.g., Sg L MH, Tp F UAE; ABTS assay: e.g., To L UAE; FRAP assay: Sg L MH, To F MH) as well as the content of: chlorophyll (e.g., To F UAE, To L MH), vitamin C (e.g., Hp H MH), total phenolic compounds (e.g., Tp F MH), macroelements in roots (N: e.g., Vo R UAE; P: e.g., Tp F MH; K: e.g., Sg L UAE; Ca: e.g., Ur L UAE; Mg: e.g., Sg L UAE; S: e.g., To L MH), microelements in leaves rosette (Fe: e.g., To F MH; Cu: e.g., Vo R UAE; Zn: e.g., Vo R UAE; Mn: e.g., To L MH; Ni: e.g., Sg L MH, Vo R MH), microelements in roots (Fe: e.g., To L UAE; Cu: e.g., To F UAE; Zn: e.g., Sg L UAE; Mn: e.g., Tp F UAE; Ni: e.g., Ur L MH), volatile compounds (e.g., limonene: e.g., Ur L UAE), fatty acids (e.g., 9, 12-hexadecadienoic acid (methyl ester): e.g., Hp H MH, Tp F MH, To F MH). In most cases, obtained bio-products lowered the content of toxic elements (Cd and Pb) in celeriac leaves and roots. They can also be used to increase (e.g., To L MH (in leaves)) or decrease (e.g., Vo R MH (in leaves); Ur L MH (in roots)) the content of nitrates. Tested botanical extracts should be used according to need.

The obtained formulations were convenient to use and effective at low concentrations. They delivered more bioavailable compounds and increased the droplet adhesion on leaves’ surface, elongated the time of wetting the leaf surface, enhanced transport and absorption of nutrients in the plant under field trials. Botanical extracts might be considered as a rich source of bioactive substances, cheap and promising strategy for achieving high yields of nutritious food and at the same time not causing the negative impact on the environment and human health. They could play a significant role in agriculture by making the crop management practices more sustainable and simultaneously to improve quality of food. However, there is still a need to improve an understanding of the mode of action of bio-products, their chemical composition and optimal usage (dose and timing) as well as their biochemical influence on plant reactions.

## Figures and Tables

**Table 1:** Effect of the foliar application of botanical extracts on the fresh weight of leaves rosette and root and the total yield of celeriac (N = 3 *, mean ± SD).

Treatment	Root Diameter < 5 cm		Root Diameter 5–9 cm		Root Diameter 9–13 cm		Total Yield		Total Yield Minus Non-Marketable Yield
	Leaves Rosette	Root	Leaves Rosette	Root	Leaves Rosette	Root	Leaves Rosette	Root	
	g						t·ha^−1^		%
CS	77.14 ± 10.50	129.29 ± 1.20	332.49 ± 71.74	368.75 ± 68.27	0	0	22.76 ± 4.57	27.67 ± 4.41	100.00
CF	76.90 ± 13.80	76.90 ± 13.80	281.79 ± 57.72	270.63 ± 32.90	226.66 ± 62.58 ^a^	586.67 ± 133.35 ^a^	18.67 ± 3.91	30.95 ± 5.91	95.89
CB	95.00 ± 12.25	90.00 ± 16.33	261.21 ± 39.15	312.18 ± 14.65	232.00 ± 58.79 ^a^	480.00 ± 130.64 ^a^	19.67 ± 3.40	30.62 ± 5.85	98.70
Hp H UAE	102.86 ± 12.99	130.48 ± 21.17	204.79 ± 41.13	255.80 ± 59.18	0	0	17.09 ± 3.01	21.46 ± 4.46	100.00
Hp H MH	133.34 ± 21.76	103.33 ± 6.83	274.69 ± 60.59	303.55 ± 68.34	475.00 ± 93.90 ^a,b,c^	850.00 ± 122.47 ^a,b,c^	32.70 ± 6.53 ^b,c^	46.55 ± 7.32 ^a,b,c^	100.00
Sg L UAE	90.00 ± 12.25	107.08 ± 12.92	275.55 ± 58.98	307.13 ± 68.39	306.69 ± 77.81 ^a^	473.34 ± 46.34 ^a^	24.90 ± 5.52	32.87 ± 4.73	100.00
Sg L MH	75.00 ± 12.25	66.67 ± 5.44 ^a^	256.38 ± 19.11	330.53 ± 75.13	0	0	15.24 ± 1.34	16.19 ± 3.14 ^b,c^	98.67
To F UAE	194.67 ± 46.08 ^a,b,c^	86.67 ± 10.50	284.52 ± 10.72	322.21 ± 24.36	0	0	20.46 ± 2.28	21.56 ± 2.12	95.16
To F MH	103.33 ± 8.06	121.00 ± 19.20	277.13 ± 43.37	294.76 ± 31.48	225.00 ± 57.15 ^a^	485.00 ± 28.58 ^a^	22.42 ± 4.02	33.36 ± 2.94	100.00
To L UAE	102.78 ± 15.77	125.00 ± 17.69	250.76 ± 50.10	286.54 ± 55.10	397.15 ± 83.41 ^a^	508.57 ± 73.95 ^a^	21.41 ± 4.20	26.67 ± 4.26	98.55
To L MH	91.56 ± 16.69	124.00 ± 19.80	292.06 ± 51.92	349.31 ± 51.99	450.00 ± 97.98 ^a,b,c^	726.67 ± 69.49 ^a^	30.87 ± 6.17 ^b^	44.44 ± 5.23 ^a,b,c^	100.00
Tp F UAE	148.20 ± 26.43 ^a,b^	167.91 ± 24.33 ^b,c^	200.88 ± 42.22	265.48 ± 55.44	0	0	15.27 ± 2.81	19.38 ± 3.26	95.88
Tp F MH	115.00 ± 12.25	160.33 ± 5.17 ^b,c^	242.37 ± 39.80	273.34 ± 25.79	316.00 ± 62.05 ^a^	444.00 ± 86.55 ^a^	24.94 ± 4.23	32.51 ± 4.35	100.00
Ur L UAE	123.17 ± 23.46	142.70 ± 8.08 b	224.00 ± 45.14	270.04 ± 58.95	0	0	13.60 ± 2.62	20.47 ± 3.24	98.72
Ur L MH	94.22 ± 5.59	113.33 ± 9.98	248.01 ± 11.67	277.22 ± 7.74	262.50 ± 63.28 ^a^	475.00 ± 102.06 ^a^	22.40 ± 2.98	32.06 ± 4.44	100.00
Vo R UAE	93.50 ± 14.29	128.80 ± 19.76	278.41 ± 27.94	316.38 ± 39.61	0	0	20.66 ± 2.35	24.73 ± 3.30	100.00
Vo R MH	79.56 ± 12.24	116.44 ± 10.89	203.33 ± 39.95	253.11 ± 38.49	0	0	13.07 ± 2.24	14.43 ± 1.92 ^b,c^	97.30

(a) Statistically significant differences (*p* < 0.05) between the control group (C) and extracts. (b) Statistically significant differences (*p* < 0.05) between water formulation (CF) and extracts. (c) Statistically significant differences (*p* < 0.05) between commercial biostimulant (CB) and extracts. Abbreviations: UAE, ultrasound assisted extraction; MH, mechanical homogenisation; Hp H, *Hypericum perforatum* L. (St. John’s wort, herb); Sg L, *Solidago gigantea* Ait. (giant goldenrod, leaf); To F, To L, *Taraxacum officinale* (L.) Weber ex F.H. Wigg (common dandelion, flower, leaf); Tp F, *Trifolium pratense* L. (red clover, flower); Ur L, *Urtica dioica* L. (nettle, leaf); Vo R, *Valeriana officinalis* L. (valerian, root). * Three replications (plots), each consisted of 32 plants.

**Table 2:** Effect of the foliar application of botanical extracts on the *L*, *a*, *b* values of celeriac leaves (N = 10, mean ± SD).

Treatment	1st Term of Leaves Rosette Collection			2nd Term of Leaves Rosette Collection		
	*L*	*a*	*b*	*L*	*a*	*b*
C	38.33 ± 0.67	−7.66 ± 0.51	12.23 ± 0.71	37.79 ± 1.49	−6.59 ± 0.61	12.33 ± 1.19
CF	38.72 ± 1.95	−7.06 ± 0.31	11.49 ± 1.45	36.81 ± 0.53	−5.30 ± 0.72	9.46 ± 0.73
CB	37.96 ± 1.45	−7.00 ± 0.31	11.14 ± 0.67	36.74 ± 0.61	−5.79 ± 0.53	10.77 ± 2.06
Hp H UAE	37.53 ± 1.40	−7.65 ± 0.30	12.15 ± 0.89	38.28 ± 0.33	−6.24 ± 0.23	10.32 ± 0.48
Hp H MH	37.86 ± 1.00	−7.01 ± 0.54	10.39 ± 0.98	35.34 ± 2.33	−6.64 ± 0.12	10.90 ± 0.21
Sg L UAE	38.63 ± 1.53	−7.10 ± 0.91	10.99 ± 1.70	35.43 ± 1.48	−4.54 ± 0.21 ^a^	6.56 ± 0.15 ^a^
Sg L MH	38.09 ± 0.88	−7.25 ± 0.41	11.87 ± 1.45	37.12 ± 1.84	−5.37 ± 0.77	8.36 ± 1.45
To F UAE	37.69 ± 1.36	−7.49 ± 0.33	11.93 ± 0.98	37.41 ± 0.37	−5.41 ± 0.29	10.30 ± 1.15
To F MH	37.32 ± 1.02	−6.77 ± 0.51 ^a^	9.93 ± 0.59 ^a^	36.50 ± 0.35	−6.02 ± 0.17	10.40 ± 0.44
To L UAE	38.52 ± 0.80	−7.33 ± 0.42	11.91 ± 1.28	34.38 ± 0.45	−5.03 ± 0.16	8.28 ± 0.46
To L MH	38.24 ± 0.43	−6.90 ± 0.17	10.95 ± 0.61	35.06 ± 0.49	−4.62 ± 0.09 ^a^	5.46 ± 0.65 ^a,c^
Tp F UAE	38.31 ± 0.93	−7.36 ± 0.44	11.85 ± 1.10	35.78 ± 0.03	−4.31 ± 0.01 ^a^	8.35 ± 0.02
Tp F MH	37.34 ± 1.05	−7.50 ± 0.25	11.09 ± 0.71	36.16 ± 1.09	−5.27 ± 0.45	8.55 ± 1.80
Ur L UAE	38.66 ± 1.38	−7.28 ± 0.38	11.46 ± 1.07	36.49 ± 0.14	−5.14 ± 0.49	8.69 ± 1.37
Ur L MH	38.42 ± 0.48	−6.93 ± 0.34	10.46 ± 0.97	37.16 ± 1.23	−5.20 ± 0.49	9.92 ± 1.57
Vo R UAE	39.39 ± 1.39	−6.97 ± 0.69	10.98 ± 1.78	37.93 ± 0.50	−6.26 ± 0.31	10.76 ± 1.54
Vo R MH	38.28 ± 0.53	−7.14 ± 0.55	11.42 ± 0.81	37.26 ± 1.30	−5.70 ± 0.73	11.63 ± 1.81

(a) Statistically significant differences (*p* < 0.05) between the control group (C) and extracts. (c) Statistically significant differences (*p* < 0.05) between commercial biostimulant (CB) and extracts. 1st term of leaves rosette collection –samples taken for analyses 7 days after the second spraying. 2nd term of leaves rosette collection—samples taken for analyses after harvesting. The *L* value indicates the level of light (numbers from 51 to 100) or dark (numbers from 0 to 50). The *a* value indicates redness (positive number) or greenness (negative number). The *b* value indicates yellowness (positive number) or blueness (negative number). Abbreviations: UAE, ultrasound assisted extraction; MH, mechanical homogenisation; Hp H, *Hypericum perforatum* L. (St. John’s wort, herb); Sg L, *Solidago gigantea* Ait. (giant goldenrod, leaf); To F, To L, *Taraxacum officinale* (L.) Weber ex F.H. Wigg (common dandelion, flower, leaf); Tp F, *Trifolium pratense* L. (red clover, flower); Ur L, *Urtica dioica* L. (nettle, leaf); Vo R, *Valeriana officinalis* L. (valerian, root).

**Table 3:** Effect of the foliar application of botanical extracts on the macroelements content of celeriac leaves and roots (N = 3, mean ± SD).

Treatment	N		P		K		Ca		Mg		S	
	Leaves	Roots	Leaves	Roots	Leaves	Roots	Leaves	Roots	Leaves	Roots	Leaves	Roots
	g·kg^−1^ DW											
C	31.25 *±* 1.56	26.30 *±* 1.32	1.88 *±* 0.09 ^c^	5.41 *±* 0.27 ^b^	34.15 *±* 1.71 ^b,c^	54.45 *±* 2.72	28.10 *±* 1.41 ^b,c^	6.45 *±* 0.32	1.92 *±* 0.10	1.98 *±* 0.10	16.68 *±* 0.83 ^c^	1.29 *±* 0.06 ^c^
CF	30.75 *±* 1.54	27.60 *±* 1.38	1.94 *±* 0.10 ^c^	6.81 *±* 0.34 ^a,c^	28.26 *±* 1.41 ^a,c^	58.33 *±* 2.92 ^c^	33.37 *±* 1.67 ^a,c^	6.92 *±* 0.35	2.01 *±* 0.10	2.32 *±* 0.12 ^c^	18.68 *±* 0.93 ^c^	1.39 *±* 0.07 ^c^
CB	32.63 *±* 1.63	27.05 *±* 1.35	1.38 *±* 0.07 ^a,b^	5.41 *±* 0.27 ^b^	23.08 *±* 1.15 ^a,b^	45.93 *±* 2.30 ^b^	10.23 *±* 0.51 ^a,b^	6.03 *±* 0.30	1.97 *±* 0.10	1.89 *±* 0.09 ^b^	13.65 *±* 0.68 ^a,b^	1.00 *±* 0.05 ^a,b^
Hp H UAE	30.88 *±* 1.54	26.35 *±* 1.32	0.97 *±* 0.05 ^a,b,c^	5.84 *±* 0.29 ^b^	24.80 *±* 1.24 ^a^	51.02 *±* 2.55	10.32 *±* 0.52 ^a,b^	6.83 *±* 0.34	1.58 *±* 0.08 ^a,b,c^	2.37 *±* 0.12 ^a,c^	18.85 *±* 0.94 ^c^	1.39 *±* 0.07 ^c^
Hp H MH	30.75 *±* 1.54	27.55 *±* 1.38	1.41 *±* 0.07 ^a,b^	5.75 *±* 0.29 ^b^	25.01 *±* 1.25 ^a^	60.81 *±* 3.04 ^c^	9.79 *±* 0.49 ^a,b^	6.93 *±* 0.35	1.78 *±* 0.09	2.39 *±* 0.12 ^a,c^	20.85 *±* 1.04 ^a,c^	1.76 *±* 0.09 ^a,b,c^
Sg L UAE	30.00 *±* 1.50	27.20 *±* 1.36	1.19 *±* 0.06 ^a,b^	6.66 *±* 0.33 ^a,c^	23.70 *±* 1.18 ^a,b^	68.94 *±* 3.45 ^a,b,c^	9.76 *±* 0.49 ^a,b^	6.82 *±* 0.34	1.88 *±* 0.09	2.64 *±* 0.13 ^a,c^	19.23 *±* 0.96 ^c^	1.45 *±* 0.07 ^c^
Sg L MH	28.95 *±* 1.45	26.50 *±* 1.33	1.28 *±* 0.06 ^a,b^	5.88 *±* 0.29 ^b^	25.35 *±* 1.27 ^a^	51.27 *±* 2.56	39.46 *±* 1.97 ^a,b,c^	6.51 *±* 0.33	1.87 *±* 0.09	2.20 *±* 0.11	20.51 *±* 1.03 ^a,c^	1.24 *±* 0.06 ^c^
To F UAE	30.13 *±* 1.51	25.50 *±* 1.28	1.28 *±* 0.06 ^a,b^	6.00 *±* 0.30	27.65 *±* 1.38 ^a,c^	56.00 *±* 2.80 ^c^	39.58 *±* 1.98 ^a,b,c^	6.20 *±* 0.31	1.83 *±* 0.09	1.99 *±* 0.10	19.63 *±* 0.98 ^a,c^	1.36 *±* 0.07 ^c^
To F MH	28.70 *±* 1.44	25.50 *±* 1.28	1.13 *±* 0.06 ^a,b,c^	5.41 *±* 0.27 ^b^	21.10 *±* 1.06 ^a,b^	51.11 *±* 2.56	10.11 *±* 0.51 ^a,b^	6.57 *±* 0.33	1.77 *±* 0.09	1.93 *±* 0.10 ^b^	19.65 *±* 0.98 ^a,c^	1.33 *±* 0.07 ^c^
To L UAE	31.25 *±* 1.56	26.20 *±* 1.31	1.28 *±* 0.06 ^a,b^	6.50 *±* 0.33 ^a,c^	25.51 *±* 1.28 ^a^	59.30 *±* 2.96 ^c^	9.76 *±* 0.49 ^a,b^	6.93 *±* 0.35	1.82 *±* 0.09	2.51 *±* 0.13 ^a,c^	17.25 *±* 0.86 ^c^	1.52 *±* 0.08 ^a,c^
To L MH	27.60 *±* 1.38 ^c^	26.15 *±* 1.31	1.03 *±* 0.05 ^a,b,c^	6.25 *±* 0.31	20.21 *±* 1.01 ^a,b^	59.30 *±* 2.96 ^c^	10.84 *±* 0.54 ^a,b^	6.70 *±* 0.33	1.82 *±* 0.09	2.44 *±* 0.12 ^a,c^	24.94 *±* 1.25 ^a,b,c^	1.76 *±* 0.09 ^a,b,c^
Tp F UAE	30.38 *±* 1.52	25.95 *±* 1.30	1.38 *±* 0.07 ^a,b^	6.19 *±* 0.31	23.76 *±* 1.19 ^a,b^	57.33 *±* 2.87 ^c^	9.69 *±* 0.48 ^a,b^	6.92 *±* 0.35	1.68 *±* 0.08 ^b,c^	2.60 *±* 0.13 ^a,c^	19.00 *±* 0.95 ^c^	1.62 *±* 0.08 ^a,b,c^
Tp F MH	31.63 *±* 1.58	27.45 *±* 1.37	1.13 *±* 0.06 ^a,b,c^	6.88 *±* 0.34 ^a,c^	20.86 *±* 1.04 ^a,b^	59.56 *±* 2.98 ^c^	10.38 *±* 0.52 ^a,b^	6.68 *±* 0.33	1.64 *±* 0.08 ^a,b,c^	2.53 *±* 0.13 ^a,c^	22.46 *±* 1.12 ^a,b,c^	1.15 *±* 0.06 ^b^
Ur L UAE	28.60 *±* 1.43	26.15 *±* 1.31	1.19 *±* 0.06 ^a,b^	6.41 *±* 0.32 ^a,c^	25.22 *±* 1.26 ^a^	65.67 *±* 3.28 ^a,c^	9.19 *±* 0.46 ^a,b^	7.01 *±* 0.35	1.82 *±* 0.09	2.51 *±* 0.13 ^a,c^	17.93 *±* 0.90 ^c^	1.26 *±* 0.06 ^c^
Ur L MH	28.65 *±* 1.43	24.43 *±* 1.22	1.75 *±* 0.09 ^c^	6.19 *±* 0.31	28.65 *±* 1.43 ^a,c^	51.43 *±* 2.57	36.04 *±* 1.80 ^a,c^	6.87 *±* 0.34	1.84 *±* 0.09	2.35 *±* 0.12 ^a,c^	16.05 *±* 0.80	1.46 *±* 0.07 ^c^
Vo R UAE	32.75 *±* 1.64	27.50 *±* 1.38	1.56 *±* 0.08 ^a,b^	5.34 *±* 0.27 ^b^	31.50 *±* 1.58 ^c^	58.75 *±* 2.94 ^b^	9.58 *±* 0.48 ^a,b^	6.93 *±* 0.35	2.01 *±* 0.10	2.26 *±* 0.11 ^c^	14.45 *±* 0.72 ^b^	1.45 *±* 0.07 ^c^
Vo R MH	32.25 *±* 1.61	25.85 *±* 1.29	1.47 *±* 0.07 ^a,b^	5.31 *±* 0.27 ^b^	28.86 *±* 1.44 ^a,c^	51.42 *±* 2.57	34.75 *±* 1.74 ^a,c^	6.26 *±* 0.31	1.89 *±* 0.09	1.96 *±* 0.10 ^b^	16.18 *±* 0.81	1.34 *±* 0.07 ^c^

(a) Statistically significant differences (*p* < 0.05) between the control group (C) and extracts. (b) Statistically significant differences (*p* < 0.05) between water formulation (CF) and extracts. (c) Statistically significant differences (*p* < 0.05) between commercial biostimulant (CB) and extracts. Abbreviations: UAE, ultrasound assisted extraction; MH, mechanical homogenisation; Hp H, *Hypericum perforatum* L. (St. John’s wort, herb); Sg L, *Solidago gigantea* Ait. (giant goldenrod, leaf); To F, To L, *Taraxacum officinale* (L.) Weber ex F.H. Wigg (common dandelion, flower, leaf); Tp F, *Trifolium pratense* L. (red clover, flower); Ur L, *Urtica dioica* L. (nettle, leaf); Vo R, *Valeriana officinalis* L. (valerian, root).

**Table 4:** Effect of the foliar application of botanical extracts on the microelements and toxic elements content of celeriac leaves and roots (N = 3, mean ± SD).

Treatment	Fe		Cu		Zn		Mn		Ni		Cd		Pb	
	Leaves	Roots	Leaves	Roots	Leaves	Roots	Leaves	Roots	Leaves	Roots	Leaves	Roots	Leaves	Roots
	mg·kg^−1^ DW													
C	262.13 *±* 13.11 ^c^	297.88 *±* 14.89	7.38 *±* 0.37	9.26 *±* 0.46 ^c^	49.21 *±* 2.46	46.23 *±* 2.31 ^c^	57.25 *±* 2.86 ^c^	34.00 *±* 1.70 ^b^	4.29 *±* 0.21 ^b,c^	6.70 *±* 0.34 ^c^	0.29 *±* 0.01 ^b^	0.54 *±* 0.03 ^c^	3.24 *±* 0.16 ^b,c^	2.88 *±* 0.14 ^b,c^
CF	318.75 *±* 15.94 ^c^	323.38 *±* 16.17 ^c^	7.81 *±* 0.39	8.10 *±* 0.41	53.84 *±* 2.69	49.30 *±* 2.47 ^c^	53.00 *±* 2.65 ^c^	42.25 *±* 2.11 ^a,c^	5.90 *±* 0.30 ^a,c^	7.68 *±* 0.38 ^c^	0.14 *±* 0.01 ^a,c^	0.48 *±* 0.02 ^c^	1.31 *±* 0.07 ^a,c^	1.47 *±* 0.07 ^a,c^
CB	475.25 *±* 23.76 ^a,b^	264.88 *±* 13.24 ^b^	7.40 *±* 0.37	7.33 *±* 0.37 ^a^	55.64 *±* 2.78	37.81 *±* 1.89 ^a,b^	70.88 *±* 3.54 ^a,b^	33.75 *±* 1.69 ^b^	9.55 *±* 0.48 ^a,b^	5.36 *±* 0.27 ^a,b^	0.25 *±* 0.01 ^b^	0.34 *±* 0.02 ^a,b^	1.77 *±* 0.09 ^a,b^	1.13 *±* 0.06 ^a,b^
Hp H UAE	448.88 *±* 22.44 ^a,b^	345.00 *±* 17.25 ^c^	7.11 *±* 0.36	7.98 *±* 0.40 ^a^	50.74 *±* 2.54	47.86 *±* 2.39 ^c^	73.50 *±* 3.68 ^a,b^	43.75 *±* 2.19 ^a,c^	7.01 *±* 0.35 ^a,c^	6.71 *±* 0.34 ^c^	0.46 *±* 0.02 ^a,b,c^	0.64 *±* 0.03 ^a,b,c^	3.58 *±* 0.18 ^b,c^	2.94 *±* 0.15 ^b,c^
Hp H MH	336.63 *±* 16.83 ^a,c^	418.25 *±* 20.91 ^a,b,c^	7.40 *±* 0.37	9.29 *±* 0.46 ^c^	51.88 *±* 2.59	42.41 *±* 2.12	71.00 *±* 3.55 ^a,b^	30.88 *±* 1.54 ^b^	6.48 *±* 0.32 ^a,c^	7.88 *±* 0.39 ^a,c^	0.33 *±* 0.02 ^a,c^	0.51 *±* 0.03 ^c^	2.22 *±* 0.11 ^a,b,c^	1.00 *±* 0.05 ^a,b^
Sg L UAE	533.88 *±* 26.69 ^a,b^	282.13 *±* 14.11	7.03 *±* 0.35	7.76 *±* 0.39 ^a^	56.19 *±* 2.81	60.50 *±* 3.03 ^a,b,c^	71.50 *±* 3.58 ^a,b^	40.50 *±* 2.03 ^a,c^	5.76 *±* 0.29 ^a,c^	4.74 *±* 0.24 ^a,b^	0.41 *±* 0.02 ^a,b,c^	0.76 *±* 0.04 ^a,b,c^	4.09 *±* 0.20 ^a,b,c^	2.91 *±* 0.15 ^b,c^
Sg L MH	506.00 *±* 25.30 ^a,b^	342.13 *±* 17.11 ^c^	6.99 *±* 0.35	8.40 *±* 0.42	47.43 *±* 2.37	40.41 *±* 2.02 ^b^	74.00 *±* 3.70 ^a,b^	39.00 *±* 1.95	13.06 *±* 0.65 ^a,b,c^	3.93 *±* 0.20 ^a,b,c^	0.35 *±* 0.02 ^a,b,c^	0.46 *±* 0.02 ^c^	2.63 *±* 0.13 ^a,b,c^	2.48 *±* 0.12 ^a,b,c^
To F UAE	347.75 *±* 17.39 ^a,c^	283.88 *±* 14.19	7.58 *±* 0.38	9.56 *±* 0.48 ^a,b^	56.00 *±* 2.80	47.93 *±* 2.40 ^c^	69.63 *±* 3.48 ^a,b^	37.63 *±* 1.88	6.23 *±* 0.31 ^a,c^	5.70 *±* 0.29 ^b^	0.28 *±* 0.01 ^b^	0.46 *±* 0.02 ^c^	2.67 *±* 0.13 ^a,b,c^	1.99 *±* 0.10 ^a,b,c^
To F MH	623.75 *±* 31.19 ^a,b,c^	284.25 *±* 14.21	7.81 *±* 0.39	8.46 *±* 0.42	55.55 *±* 2.78	42.68 *±* 2.13	82.50 *±* 4.13 ^a,b,c^	38.63 *±* 1.93	7.26 *±* 0.36 ^a,b,c^	8.66 *±* 0.43 ^a,c^	0.38 *±* 0.02 ^a,b,c^	0.43 *±* 0.02 ^a,c^	2.36 *±* 0.12 ^a,b,c^	1.08 *±* 0.05 ^a,b^
To L UAE	485.25 *±* 24.26 ^a,b^	452.13 *±* 22.61 ^a,b,c^	7.56 *±* 0.38	9.13 *±* 0.46 ^c^	55.43 *±* 2.77	51.15 *±* 2.56 ^c^	72.63 *±* 3.63 ^a,b^	45.00 *±* 2.25 ^a,c^	7.88 *±* 0.39 ^a,b,c^	5.33 *±* 0.27 ^a,b^	0.40 *±* 0.02 ^a,b,c^	0.65 *±* 0.03 ^a,b,c^	3.80 *±* 0.19 ^a,b,c^	3.28 *±* 0.16 ^a,b,c^
To L MH	509.50 *±* 25.48 ^a,b^	410.50 *±* 20.53 ^a,b,c^	7.14 *±* 0.36	8.56 *±* 0.43	53.76 *±* 2.69	43.41 *±* 2.17	92.38 *±* 4.62 ^a,b,c^	42.88 *±* 2.14 ^a,c^	6.81 *±* 0.34 ^a,c^	7.16 *±* 0.36 ^c^	0.49 *±* 0.02 ^a,b,c^	0.40 *±* 0.02 ^a^	3.08 *±* 0.15 ^b,c^	1.67 *±* 0.08 ^a,c^
Tp F UAE	503.75 *±* 25.19 ^a,b^	405.63 *±* 20.28 ^a,b,c^	7.74 *±* 0.39	8.01 *±* 0.40	53.83 *±* 2.69	50.50 *±* 2.53 ^c^	69.50 *±* 3.48 ^a,b^	45.38 *±* 2.27 ^a,c^	7.54 *±* 0.38 ^a,b,c^	6.89 *±* 0.34 ^c^	0.38 *±* 0.02 ^a,b,c^	0.60 *±* 0.03 ^b,c^	3.12 *±* 0.16 ^b,c^	2.43 *±* 0.12 ^a,b,c^
Tp F MH	467.50 *±* 23.38 ^a,b^	288.38 *±* 14.42	7.51 *±* 0.38	8.19 *±* 0.41	54.04 *±* 2.70	42.65 *±* 2.13	88.63 *±* 4.43 ^a,b,c^	38.63 *±* 1.93	7.29 *±* 0.36 ^a,b,c^	7.13 *±* 0.36 ^c^	0.43 *±* 0.02 ^a,b,c^	0.38 *±* 0.02 ^a,b^	1.27 *±* 0.06 ^a,c^	1.58 *±* 0.08 ^a,c^
Ur L UAE	486.00 *±* 24.30 ^a,b^	400.63 *±* 20.03 ^a,b,c^	7.34 *±* 0.37	8.21 *±* 0.41	52.65 *±* 2.63	52.63 *±* 2.63 ^c^	71.88 *±* 3.59 ^a,b^	43.63 *±* 2.18 ^a,c^	3.61 *±* 0.18 ^b,c^	7.08 *±* 0.35 ^c^	0.38 *±* 0.02 ^a,b,c^	0.61 *±* 0.03 ^b,c^	3.68 *±* 0.18 ^a,b,c^	1.84 *±* 0.09 ^a,b,c^
Ur L MH	347.88 *±* 17.39 ^a,c^	382.63 *±* 19.13 ^a,b,c^	7.56 *±* 0.38	8.04 *±* 0.40	49.89 *±* 2.49	43.94 *±* 2.20	56.50 *±* 2.83 ^c^	42.50 *±* 2.13 ^a,c^	5.89 *±* 0.29 ^a,c^	13.63 *±* 0.68 ^a,b,c^	0.20 *±* 0.01 ^a,b^	0.41 *±* 0.02 ^a^	1.30 *±* 0.07 ^a,c^	1.44 *±* 0.07 ^a^
Vo R UAE	329.75 *±* 16.49 ^a,c^	409.13 *±* 20.46 ^a,b,c^	8.10 *±* 0.41	7.26 *±* 0.36 ^a^	60.70 *±* 3.04 ^a^	49.54 *±* 2.48 ^c^	61.38 *±* 3.07	43.50 *±* 2.18 ^a,c^	5.46 *±* 0.27 ^a,c^	7.11 *±* 0.36 ^c^	0.41 *±* 0.02 ^a,b,c^	0.49 *±* 0.02 ^c^	3.86 *±* 0.19 ^a,b,c^	3.05 *±* 0.15 ^b,c^
Vo R MH	302.00 *±* 15.10 ^c^	268.88 *±* 13.44 ^b^	7.79 *±* 0.39	8.11 *±* 0.41	58.78 *±* 2.94 ^a^	46.31 *±* 2.32 ^c^	60.63 *±* 3.03	38.25 *±* 1.91	13.06 *±* 0.65 ^a,b,c^	4.66 *±* 0.23 ^a,b^	0.34 *±* 0.02 ^b,c^	0.49 *±* 0.02 ^c^	1.83 *±* 0.09 ^a,b^	1.14 *±* 0.06 ^a^

(a) Statistically significant differences (*p* < 0.05) between the control group (C) and extracts. (b) Statistically significant differences (*p* < 0.05) between water formulation (CF) and extracts. (c) Statistically significant differences (*p* < 0.05) between commercial biostimulant (CB) and extracts. Abbreviations: UAE, ultrasound assisted extraction; MH, mechanical homogenisation; Hp H, *Hypericum perforatum* L. (St. John’s wort, herb); Sg L, *Solidago gigantea* Ait. (giant goldenrod, leaf); To F, To L, *Taraxacum officinale* (L.) Weber ex F.H. Wigg (common dandelion, flower, leaf); Tp F, *Trifolium pratense* L. (red clover, flower); Ur L, *Urtica dioica* L. (nettle, leaf); Vo R, *Valeriana officinalis* L. (valerian, root).

**Table 5:** Effect of the foliar application of botanical extracts on the volatile compounds profile (amount of single component calculated as percent (%) of whole GC-MS chromatogram area) of celeriac leaves (N = 3, mean ± SD).

**Treatment**	**Hexanal**	**2-hexenal**	**α** **-pinene**	**Camphene**	**Sabinene**	**β-pinene**	**1-octen-3-ol**	**β-myrcene**	**Octanal**	**p-cymene**
RT, min	4.545	6.055	9.225	9.900	11.190	11.295	11.640	12.430	12.890	14.050
C	0.020 *±* 0.002	0.063 *±* 0.007	0.119 *±* 0.008	0.013 *±* 0.003	0.072 *±* 0.004	0.160 *±* 0.012	0.037 *±* 0.003 ^b,c^	24.827 *±* 0.230 ^b,c^	0.019 *±* 0.002	0.202 *±* 0.007
CF	0.021 *±* 0.001	0.051 *±* 0.001	0.108 *±* 0.001	0.010 *±* 0.003	0.076 *±* 0.002	0.164 *±* 0.000	0.028 *±* 0.001 ^a^	29.385 *±* 0.114 ^a^	0.015 *±* 0.002	0.203 *±* 0.002
CB	0.024 *±* 0.002	0.042 *±* 0.005	0.105 *±* 0.003	0.013 *±* 0.003	0.079 *±* 0.001	0.164 *±* 0.004	0.026 *±* 0.000 ^a^	29.894 *±* 0.536 ^a^	0.017 *±* 0.005	0.205 *±* 0.011
Hp H UAE	0.024 *±* 0.002	0.050 *±* 0.009	0.114 *±* 0.004	0.011 *±* 0.002	0.071 *±* 0.003	0.174 *±* 0.006	0.024 *±* 0.002 ^a^	30.259 *±* 0.550 ^a^	0.015 *±* 0.004	0.170 *±* 0.007
Hp H MH	0.018 *±* 0.002	0.056 *±* 0.005	0.125 *±* 0.003 ^b,c^	0.016 *±* 0.000	0.067 *±* 0.002 ^c^	0.166 *±* 0.005	0.028 *±* 0.001 ^a^	25.024 *±* 0.342 ^b,c^	0.017 *±* 0.002	0.186 *±* 0.013
Sg L UAE	0.016 *±* 0.002	0.030 *±* 0.005 ^a^	0.101 *±* 0.005 ^a^	0.009 *±* 0.002	0.077 *±* 0.004	0.144 *±* 0.006	0.016 *±* 0.002 ^a,b,c^	29.635 *±* 0.496 ^a^	0.016 *±* 0.003	0.124 *±* 0.025 ^a,b,c^
Sg L MH	0.018 *±* 0.002	0.026 *±* 0.004 ^a,b^	0.089 *±* 0.003 ^a,b,c^	0.010 *±* 0.002	0.078 *±* 0.002	0.164 *±* 0.006	0.014 *±* 0.002 ^a,b,c^	39.158 *±* 0.748 ^a,b,c^	0.017 *±* 0.003	0.156 *±* 0.006
To F UAE	0.022 *±* 0.003	0.046 *±* 0.008	0.109 *±* 0.003	0.010 *±* 0.002	0.078 *±* 0.001	0.179 *±* 0.005	0.025 *±* 0.002 ^a^	27.492 *±* 0.375 ^a,c^	0.019 *±* 0.003	0.193 *±* 0.022
To F MH	0.019 *±* 0.001	0.038 *±* 0.005 ^a^	0.103 *±* 0.003 ^a^	0.016 *±* 0.000	0.085 *±* 0.001 ^a^	0.191 *±* 0.005 ^a,b,c^	0.014 *±* 0.002 ^a,b,c^	38.291 *±* 0.869 ^a,b,c^	0.020 *±* 0.002	0.195 *±* 0.021
To L UAE	0.014 *±* 0.001	0.022 *±* 0.005 ^a,b^	0.092 *±* 0.004 ^a,b^	0.009 *±* 0.001	0.077 *±* 0.002	0.135 *±* 0.004 ^a,b,c^	0.019 *±* 0.001 ^a,b,c^	31.486 *±* 0.537 ^a^	0.018 *±* 0.003	0.179 *±* 0.014
To L MH	0.017 *±* 0.002	0.032 *±* 0.007 ^a^	0.094 *±* 0.003 ^a^	0.011 *±* 0.002	0.079 *±* 0.002	0.165 *±* 0.005	0.016 *±* 0.002 ^a,b,c^	36.728 *±* 0.793 ^a,b,c^	0.019 *±* 0.002	0.174 *±* 0.020
Tp F UAE	0.026 *±* 0.003	0.032 *±* 0.003 ^a^	0.097 *±* 0.002 ^a^	0.010 *±* 0.002	0.065 *±* 0.002 ^b,c^	0.201 *±* 0.004 ^a,b,c^	0.010 *±* 0.000 ^a,b,c^	38.689 *±* 0.763 ^a,b,c^	0.018 *±* 0.004	0.154 *±* 0.004
Tp F MH	0.026 *±* 0.003	0.054 *±* 0.010	0.100 *±* 0.003 ^a^	0.014 *±* 0.000	0.084 *±* 0.005 ^a^	0.178 *±* 0.009	0.015 *±* 0.002 ^a,b,c^	34.362 *±* 0.902 ^a,b,c^	0.019 *±* 0.002	0.126 *±* 0.003 ^a,b,c^
Ur L UAE	0.026 *±* 0.002 ^c^	0.039 *±* 0.008 ^a^	0.114 *±* 0.003	0.011 *±* 0.002	0.070 *±* 0.002	0.150 *±* 0.005	0.022 *±* 0.001 ^a,b^	22.787 *±* 0.238 ^b,c^	0.016 *±* 0.002	0.138 *±* 0.026 ^a,b,c^
Ur L MH	0.021 *±* 0.003	0.055 *±* 0.004	0.112 *±* 0.004	0.015 *±* 0.001	0.071 *±* 0.002	0.155 *±* 0.005	0.030 *±* 0.001 ^a^	27.867 *±* 0.500 ^a^	0.017 *±* 0.003	0.184 *±* 0.015
Vo R UAE	0.016 *±* 0.002	0.035 *±* 0.005 ^a^	0.137 *±* 0.008 ^a,b,c^	0.020 *±* 0.001 ^b^	0.067 *±* 0.003 ^c^	0.180 *±* 0.009	0.019 *±* 0.001 ^a,b,c^	33.828 *±* 0.726 ^a,b,c^	0.014 *±* 0.004	0.150 *±* 0.003
Vo R MH	0.019 *±* 0.001	0.042 *±* 0.008	0.103 *±* 0.003 ^a^	0.028 *±* 0.005 ^a,b,c^	0.094 *±* 0.004 ^a,b,c^	0.113 *±* 0.007 ^a,b,c^	0.021 *±* 0.003 ^a,b^	29.736 *±* 0.526 ^a^	0.015 *±* 0.002	0.134 *±* 0.026 ^a,b,c^
**Treatment**	**Limonene**	***Z*** **-β-ocimene**	***E*** **-β-ocimene**	**Oct-(5*Z*)-enol**	**γ-terpinene**	**Terpinolene**	**6, 7-epoxymyrcene**	**Linalool**	**Perillene**	**1-octen-3-ol, acetate**
RT, min	14.535	14.980	15.570	15.840	16.105	17.910	18.335	18.815	18.960	19.690
C	52.465 *±* 0.657	4.396 *±* 0.126 ^b,c^	0.271 *±* 0.017 ^b,c^	0.004 *±* 0.001	0.412 *±* 0.020 ^b,c^	0.005 *±* 0.001	0.010 *±* 0.000 ^b,c^	0.019 *±* 0.001	0.029 *±* 0.003	0.002 *±* 0.000 ^b,c^
CF	53.615 *±* 0.095	2.754 *±* 0.006 ^a^	0.113 *±* 0.001 ^a^	0.006 *±* 0.000	0.297 *±* 0.003 ^a^	0.004 *±* 0.000	0.021 *±* 0.001 ^a^	0.023 *±* 0.000	0.030 *±* 0.001	0.008 *±* 0.000 ^a^
CB	54.079 *±* 0.480	2.722 *±* 0.071 ^a^	0.119 *±* 0.005 ^a^	0.006 *±* 0.000	0.276 *±* 0.009 ^a^	0.004 *±* 0.001	0.023 *±* 0.001 ^a^	0.024 *±* 0.002	0.024 *±* 0.004	0.007 *±* 0.001 ^a^
Hp H UAE	50.724 *±* 0.428 ^a,b,c^	2.744 *±* 0.094 ^a^	0.124 *±* 0.005 ^a^	0.006 *±* 0.002	0.326 *±* 0.014 ^a,c^	0.004 *±* 0.000	0.017 *±* 0.001 ^a,c^	0.022 *±* 0.001	0.051 *±* 0.003 ^a,b,c^	0.004 *±* 0.000
Hp H MH	56.534 *±* 0.629 ^a,b,c^	3.550 *±* 0.097 ^a,b,c^	0.185 *±* 0.010 ^a,b,c^	0.006 *±* 0.001	0.386 *±* 0.016 ^b,c^	0.004 *±* 0.000	0.012 *±* 0.001 ^b,c^	0.019 *±* 0.001	0.028 *±* 0.005	0.004 *±* 0.000 ^b^
Sg L UAE	57.009 *±* 0.443 ^a,b,c^	1.732 *±* 0.072 ^a,b,c^	0.077 *±* 0.005 ^a,b,c^	0.007 *±* 0.000	0.178 *±* 0.008 ^a,b,c^	0.003 *±* 0.001	0.020 *±* 0.001 ^a^	0.024 *±* 0.001	0.017 *±* 0.003 ^b^	0.007 *±* 0.001 ^a^
Sg L MH	50.255 *±* 0.086 ^a,b,c^	1.239 *±* 0.052 ^a,b,c^	0.056 *±* 0.002 ^a,b,c^	0.011 *±* 0.003 ^a^	0.130 *±* 0.007 ^a,b,c^	0.004 *±* 0.001	0.036 *±* 0.001 ^a,b,c^	0.033 *±* 0.002 ^a,b,c^	0.016 *±* 0.004 ^a,b^	0.006 *±* 0.001
To F UAE	54.483 *±* 0.450 ^a^	2.685 *±* 0.086 ^a^	0.124 *±* 0.006 ^a^	0.005 *±* 0.001	0.394 *±* 0.017 ^b,c^	0.005 *±* 0.000	0.011 *±* 0.001 ^b,c^	0.024 *±* 0.001	0.021 *±* 0.004	0.003 *±* 0.001 ^b,c^
To F MH	47.071 *±* 0.088 ^a,b,c^	1.912 *±* 0.083 ^a,b,c^	0.078 *±* 0.004 ^a,b,c^	0.009 *±* 0.002	0.240 *±* 0.012 ^a,b^	0.004 *±* 0.000	0.030 *±* 0.002 ^a,b,c^	0.034 *±* 0.002 ^a,b,c^	0.033 *±* 0.002	0.008 *±* 0.003 ^a^
To L UAE	57.046 *±* 0.408 ^a,b,c^	1.590 *±* 0.074 ^a,b,c^	0.078 *±* 0.004 ^a,b,c^	0.006 *±* 0.002	0.228 *±* 0.010 ^a,b,c^	0.003 *±* 0.000	0.015 *±* 0.002 ^b,c^	0.025 *±* 0.002 ^a^	0.017 *±* 0.003 ^b^	0.006 *±* 0.000
To L MH	49.559 *±* 0.051 ^a,b,c^	1.541 *±* 0.078 ^a,b,c^	0.065 *±* 0.004 ^a,b,c^	0.009 *±* 0.002	0.232 *±* 0.012 ^a,b^	0.005 *±* 0.000	0.026 *±* 0.002 ^a^	0.028 *±* 0.001 ^a^	0.029 *±* 0.003	0.008 *±* 0.000 ^a^
Tp F UAE	42.966 *±* 0.201 ^a,b,c^	1.878 *±* 0.051 ^a,b,c^	0.078 *±* 0.004 ^a,b,c^	0.012 *±* 0.002 ^a,b,c^	0.161 *±* 0.005 ^a,b,c^	0.004 *±* 0.001	0.021 *±* 0.001 ^a^	0.037 *±* 0.001 ^a,b,c^	0.034 *±* 0.004	0.002 *±* 0.001 ^b,c^
Tp F MH	52.058 *±* 0.344 ^c^	1.708 *±* 0.065 ^a,b,c^	0.070 *±* 0.004 ^a,b,c^	0.009 *±* 0.002 ^a^	0.150 *±* 0.007 ^a,b,c^	0.003 *±* 0.000	0.030 *±* 0.002 ^a,b,c^	0.031 *±* 0.002 ^a,b,c^	0.034 *±* 0.004	0.007 *±* 0.001 ^a^
Ur L UAE	60.470 *±* 0.694 ^a,b,c^	2.842 *±* 0.101 ^a^	0.115 *±* 0.007 ^a^	0.006 *±* 0.000	0.309 *±* 0.014 ^a^	0.004 *±* 0.001	0.010 *±* 0.001 ^b,c^	0.017 *±* 0.000 ^b,c^	0.024 *±* 0.004	0.005 *±* 0.001
Ur L MH	52.989 *±* 0.416	3.412 *±* 0.123 ^a,b,c^	0.151 *±* 0.008 ^a,b,c^	0.006 *±* 0.001	0.340 *±* 0.015 ^a,c^	0.003 *±* 0.001	0.019 *±* 0.001 ^a^	0.022 *±* 0.001	0.017 *±* 0.001	0.007 *±* 0.001 ^a^
Vo R UAE	47.777 *±* 0.443 ^a,b,c^	2.475 *±* 0.080 ^a^	0.127 *±* 0.006 ^a^	0.005 *±* 0.001	0.217 *±* 0.009 ^a,b,c^	0.006 *±* 0.001	0.025 *±* 0.004 ^a^	0.028 *±* 0.002 ^a^	0.027 *±* 0.004	0.006 *±* 0.002
Vo R MH	53.331 *±* 0.584	2.620 *±* 0.352 ^a^	0.139 *±* 0.005 ^a,b^	0.005 *±* 0.000	0.272 *±* 0.011 ^a^	0.004 *±* 0.001	0.017 *±* 0.002 ^a^	0.024 *±* 0.001	0.020 *±* 0.001	0.006 *±* 0.001
**Treatment**	***trans*** **-p-mentha-2, 8-dien-1-ol**	**Myrcenol**	**butyl-2-methyl-pentanoate**	***allo*** **-ocim-(4*E*, 6*Z*)-en**	***cis*** **-p-mentha-2, 8-dien-1-ol**	***cis*** **-limonene oxide**	**5-pentylcyclohexa-1, 3-diene**	**Ipsdienol**	**Eucarvone**	***cis*** **-linalool oxide (pyranoid)**
RT, min	19.940	20.135	20.445	20.635	20.885	21.045	21.400	21.815	22.370	22.985
C	0.024 *±* 0.002 ^c^	0.006 *±* 0.000	0.007 *±* 0.000 ^b,c^	0.118 *±* 0.002 ^b,c^	0.011 *±* 0.001	0.084 *±* 0.005 ^b,c^	0.006 *±* 0.000	0.005 *±* 0.001	0.736 *±* 0.049 ^b,c^	0.023 *±* 0.001 ^c^
CF	0.028 *±* 0.001	0.005 *±* 0.001	0.016 *±* 0.001 ^a^	0.078 *±* 0.002 ^a^	0.010 *±* 0.002	0.146 *±* 0.002 ^a^	0.007 *±* 0.000	0.004 *±* 0.000	0.342 *±* 0.008 ^a^	0.029 *±* 0.002
CB	0.034 *±* 0.001 ^a^	0.006 *±* 0.000	0.014 *±* 0.001 ^a^	0.080 *±* 0.001 ^a^	0.014 *±* 0.002	0.172 *±* 0.007 ^a^	0.004 *±* 0.001	0.005 *±* 0.000	0.276 *±* 0.014 ^a^	0.034 *±* 0.001 ^a^
Hp H UAE	0.034 *±* 0.001 ^a^	0.003 *±* 0.000	0.012 *±* 0.001	0.073 *±* 0.002 ^a^	0.013 *±* 0.002	0.101 *±* 0.005 ^b,c^	0.026 *±* 0.001 ^a,b,c^	0.006 *±* 0.000	0.464 *±* 0.025 ^a,b,c^	0.018 *±* 0.001 ^b,c^
Hp H MH	0.026 *±* 0.001 ^c^	0.006 *±* 0.001	0.008 *±* 0.001 ^b,c^	0.094 *±* 0.002 ^a,b,c^	0.010 *±* 0.002	0.127 *±* 0.006 ^a,c^	0.006 *±* 0.001	0.006 *±* 0.003	0.462 *±* 0.022 ^a,b,c^	0.018 *±* 0.002 ^b,c^
Sg L UAE	0.028 *±* 0.001	0.006 *±* 0.000	0.008 *±* 0.001 ^b,c^	0.056 *±* 0.002 ^a,b,c^	0.011 *±* 0.001	0.160 *±* 0.008 ^a^	0.004 *±* 0.000	0.004 *±* 0.000	0.178 *±* 0.010 ^a,b,c^	0.028 *±* 0.001
Sg L MH	0.025 *±* 0.001 ^c^	0.005 *±* 0.001	0.009 *±* 0.001 ^b^	0.041 *±* 0.001 ^a,b,c^	0.008 *±* 0.002	0.184 *±* 0.009 ^a,b^	0.006 *±* 0.001	0.006 *±* 0.002	0.111 *±* 0.009 ^a,b,c^	0.028 *±* 0.003
To F UAE	0.031 *±* 0.002	0.006 *±* 0.001	0.009 *±* 0.001 ^b,c^	0.077 *±* 0.002 ^a^	0.013 *±* 0.002	0.109 *±* 0.005 ^b,c^	0.006 *±* 0.000	0.006 *±* 0.000	0.417 *±* 0.026 ^a,b,c^	0.020 *±* 0.003 ^b,c^
To F MH	0.028 *±* 0.003	0.006 *±* 0.001	0.014 *±* 0.001 ^a^	0.060 *±* 0.001 ^a,b,c^	0.012 *±* 0.001	0.141 *±* 0.008 ^a,c^	0.011 *±* 0.003 ^c^	0.005 *±* 0.001	0.159 *±* 0.016 ^a,b,c^	0.032 *±* 0.001 ^a^
To L UAE	0.018 *±* 0.003 ^b,c^	0.005 *±* 0.001	0.011 *±* 0.001	0.044 *±* 0.002 ^a,b,c^	0.005 *±* 0.001 ^a,c^	0.124 *±* 0.010 ^a,c^	0.004 *±* 0.001	0.005 *±* 0.000	0.211 *±* 0.012 ^a,b^	0.013 *±* 0.001 ^a,b,c^
To L MH	0.028 *±* 0.002	0.005 *±* 0.001	0.011 *±* 0.001	0.047 *±* 0.001 ^a,b,c^	0.011 *±* 0.000	0.142 *±* 0.008 ^a,c^	0.009 *±* 0.003	0.005 *±* 0.001	0.232 *±* 0.014 ^a,b^	0.024 *±* 0.000 ^c^
Tp F UAE	0.030 *±* 0.002	0.008 *±* 0.000	0.006 *±* 0.000 ^b,c^	0.059 *±* 0.000 ^a,b,c^	0.011 *±* 0.003	0.097 *±* 0.003 ^b,c^	0.008 *±* 0.002	0.006 *±* 0.000	0.202 *±* 0.010 ^a,b,c^	0.032 *±* 0.002 ^a^
Tp F MH	0.037 *±* 0.003 ^a,b^	0.009 *±* 0.002 ^b^	0.011 *±* 0.001 ^b^	0.060 *±* 0.004 ^a,b,c^	0.015 *±* 0.001	0.178 *±* 0.016 ^a,c^	0.012 *±* 0.003 ^a,c^	0.004 *±* 0.000	0.227 *±* 0.011 ^a,b^	0.033 *±* 0.001 ^a^
Ur L UAE	0.031 *±* 0.001	0.007 *±* 0.001	0.011 *±* 0.001	0.080 *±* 0.001 ^a^	0.014 *±* 0.001	0.112 *±* 0.005 ^b,c^	0.004 *±* 0.001	0.005 *±* 0.001	0.354 *±* 0.018 ^a,c^	0.024 *±* 0.002 ^c^
Ur L MH	0.032 *±* 0.001 ^a^	0.007 *±* 0.000	0.010 *±* 0.002 ^b^	0.093 *±* 0.002 ^a,b,c^	0.013 *±* 0.002	0.138 *±* 0.008 ^a,c^	0.006 *±* 0.001	0.005 *±* 0.000	0.498 *±* 0.025 ^a,b,c^	0.029 *±* 0.001
Vo R UAE	0.020 *±* 0.002 ^b,c^	0.004 *±* 0.001	0.008 *±* 0.002 ^b,c^	0.073 *±* 0.001 ^a^	0.008 *±* 0.001 ^c^	0.135 *±* 0.006 ^a,c^	0.006 *±* 0.002	0.005 *±* 0.001	0.267 *±* 0.002 ^a,b^	0.033 *±* 0.000 ^a^
Vo R MH	0.025 *±* 0.003 ^c^	0.003 *±* 0.001	0.011 *±* 0.002 ^b^	0.077 *±* 0.001 ^a^	0.008 *±* 0.002 ^c^	0.103 *±* 0.004 ^b,c^	0.006 *±* 0.000	0.005 *±* 0.001	0.379 *±* 0.017 ^a,c^	0.016 *±* 0.003 ^a,b,c^
**Treatment**	***trans*** **- β -terpineol**	***iso*** **-menthol**	**2-heptyl-furan**	**β-cyclocitral**	***cis*** **-4-caranone**	***trans*** **-carveol**	***cis*** **-p-mentha-1(7), 8-dien-2-ol**	**Carvone**	**β-cyclohomocitral**	***cis*** **-carvone oxide**
RT, min	23.610	24.550	25.200	25.430	25.900	26.440	27.235	28.015	28.880	29.355
C	0.021 *±* 0.002 ^b,c^	0.003 *±* 0.000	0.007 *±* 0.001	0.065 *±* 0.005 ^c^	0.086 *±* 0.006	0.049 *±* 0.005	0.019 *±* 0.004	0.015 *±* 0.002	0.003 *±* 0.001	0.004 *±* 0.000
CF	0.012 *±* 0.001 ^a^	0.003 *±* 0.000	0.007 *±* 0.000	0.078 *±* 0.002	0.079 *±* 0.000	0.048 *±* 0.000	0.012 *±* 0.002	0.013 *±* 0.000	0.003 *±* 0.000	0.004 *±* 0.000
CB	0.012 *±* 0.002 ^a^	0.003 *±* 0.000	0.009 *±* 0.000	0.095 *±* 0.009 ^a^	0.099 *±* 0.009	0.065 *±* 0.006	0.017 *±* 0.002	0.018 *±* 0.003	0.002 *±* 0.000	0.005 *±* 0.001
Hp H UAE	0.017 *±* 0.002	0.002 *±* 0.000	0.007 *±* 0.000	0.054 *±* 0.004 ^c^	0.057 *±* 0.006 ^a,c^	0.048 *±* 0.006	0.016 *±* 0.003	0.015 *±* 0.004	0.004 *±* 0.000	0.005 *±* 0.001
Hp H MH	0.013 *±* 0.003	0.003 *±* 0.001	0.007 *±* 0.000	0.058 *±* 0.004 ^c^	0.066 *±* 0.005 ^c^	0.057 *±* 0.005	0.019 *±* 0.004	0.016 *±* 0.003	0.005 *±* 0.001	0.003 *±* 0.001
Sg L UAE	0.010 *±* 0.001 ^a^	0.003 *±* 0.000	0.008 *±* 0.001	0.086 *±* 0.008	0.095 *±* 0.009	0.050 *±* 0.007	0.015 *±* 0.003	0.020 *±* 0.003	0.003 *±* 0.001	0.005 *±* 0.001
Sg L MH	0.008 *±* 0.001 ^a^	0.004 *±* 0.001	0.005 *±* 0.000 ^c^	0.082 *±* 0.006	0.072 *±* 0.006 ^c^	0.047 *±* 0.003	0.011 *±* 0.003	0.018 *±* 0.002	0.003 *±* 0.001	0.006 *±* 0.002
To F UAE	0.015 *±* 0.003	0.003 *±* 0.001	0.009 *±* 0.000	0.066 *±* 0.004 ^c^	0.074 *±* 0.006 ^c^	0.054 *±* 0.006	0.020 *±* 0.004	0.015 *±* 0.003	0.003 *±* 0.001	0.005 *±* 0.000
To F MH	0.012 *±* 0.002 ^a^	0.004 *±* 0.000	0.009 *±* 0.001	0.099 *±* 0.008 ^a^	0.100 *±* 0.007	0.051 *±* 0.006	0.014 *±* 0.004	0.015 *±* 0.003	0.003 *±* 0.000	0.006 *±* 0.001
To L UAE	0.007 *±* 0.000 ^a^	0.004 *±* 0.000	0.004 *±* 0.000 ^c^	0.042 *±* 0.003 ^b,c^	0.045 *±* 0.003 ^a,b,c^	0.039 *±* 0.004 ^c^	0.013 *±* 0.004	0.015 *±* 0.004	0.003 *±* 0.001	0.004 *±* 0.000
To L MH	0.011 *±* 0.002 ^a^	0.003 *±* 0.000	0.006 *±* 0.000	0.067 *±* 0.009 ^c^	0.064 *±* 0.006 ^c^	0.046 *±* 0.004	0.013 *±* 0.003	0.015 *±* 0.004	0.002 *±* 0.000	0.006 *±* 0.000
Tp F UAE	0.011 *±* 0.000 ^a^	0.003 *±* 0.000	0.006 *±* 0.001	0.080 *±* 0.006	0.084 *±* 0.007	0.053 *±* 0.005	0.011 *±* 0.003	0.015 *±* 0.003	0.003 *±* 0.000	0.007 *±* 0.002
Tp F MH	0.013 *±* 0.002 ^a^	0.003 *±* 0.000	0.011 *±* 0.002 ^a,b^	0.104 *±* 0.010 ^a^	0.105 *±* 0.006 ^b^	0.061 *±* 0.006	0.016 *±* 0.003	0.024 *±* 0.002	0.003 *±* 0.000	0.007 *±* 0.000
Ur L UAE	0.016 *±* 0.002	0.004 *±* 0.000	0.008 *±* 0.001	0.069 *±* 0.007 ^c^	0.069 *±* 0.006 ^c^	0.052 *±* 0.005	0.016 *±* 0.003	0.016 *±* 0.004	0.003 *±* 0.001	0.005 *±* 0.001
Ur L MH	0.016 *±* 0.002	0.004 *±* 0.001	0.010 *±* 0.001	0.084 *±* 0.008	0.093 *±* 0.009	0.059 *±* 0.006	0.018 *±* 0.004	0.015 *±* 0.002	0.002 *±* 0.000	0.005 *±* 0.000
Vo R UAE	0.009 *±* 0.002 ^a^	0.004 *±* 0.000	0.005 *±* 0.001 ^c^	0.102 *±* 0.010 ^a^	0.085 *±* 0.009	0.040 *±* 0.005 ^c^	0.014 *±* 0.003	0.017 *±* 0.001	0.004 *±* 0.001	0.007 *±* 0.001
Vo R MH	0.015 *±* 0.003	0.005 *±* 0.001	0.007 *±* 0.001	0.056 *±* 0.003 ^c^	0.054 *±* 0.005 ^a,b,c^	0.048 *±* 0.006	0.019 *±* 0.003	0.017 *±* 0.004	0.003 *±* 0.000	0.005 *±* 0.002
**Treatment**	**Geranial**	**2-undecanone**	***iso*** **-dihydrocarveol acetate**	***trans*** **-carvyl acetate**	**Verdoracine**	**-** **longipinene**	***cis*** **-carvyl acetate**	**Longifolene**	**α** **-cedrene**	**α** **-humulene**
RT, min	29.830	31.540	33.665	34.305	35.180	35.490	35.900	39.155	39.540	41.240
C	0.010 *±* 0.001 ^b,c^	3.411 *±* 0.223 ^b^	0.005 *±* 0.001 ^b,c^	0.078 *±* 0.003 ^c^	0.010 *±* 0.001	0.003 *±* 0.001 ^c^	0.002 *±* 0.000 ^c^	0.044 *±* 0.003 ^b,c^	0.005 *±* 0.000	0.004 *±* 0.000
CF	0.021 *±* 0.001 ^a,c^	2.522 *±* 0.036 ^a,c^	0.011 *±* 0.000 ^a^	0.082 *±* 0.002 ^c^	0.007 *±* 0.002	0.005 *±* 0.002	0.003 *±* 0.000	0.062 *±* 0.002 ^a^	0.004 *±* 0.000	0.006 *±* 0.001
CB	0.030 *±* 0.002 ^a,b^	3.637 *±* 0.220 ^b^	0.010 *±* 0.001 ^a^	0.134 *±* 0.005 ^a,b^	0.010 *±* 0.002	0.008 *±* 0.002 ^a^	0.005 *±* 0.001 ^a^	0.080 *±* 0.004 ^a^	0.003 *±* 0.001	0.008 *±* 0.000
Hp H UAE	0.022 *±* 0.000 ^a,c^	3.338 *±* 0.227 ^b^	0.009 *±* 0.001	0.102 *±* 0.006 ^a,b,c^	0.011 *±* 0.001	0.005 *±* 0.000	0.003 *±* 0.000	0.140 *±* 0.007 ^a^^,^^b,c^	0.005 *±* 0.000	0.011 *±* 0.000 ^a,b^
Hp H MH	0.015 *±* 0.003 ^c^	3.831 *±* 0.219 ^b^	0.009 *±* 0.001	0.129 *±* 0.004 ^a,b^	0.009 *±* 0.002	0.005 *±* 0.001	0.004 *±* 0.000	0.072 *±* 0.003 ^a^	0.005 *±* 0.001	0.005 *±* 0.002
Sg L UAE	0.026 *±* 0.001 ^a^	2.417 *±* 0.174 ^a,c^	0.007 *±* 0.000	0.080 *±* 0.004 ^c^	0.007 *±* 0.001	0.008 *±* 0.002	0.003 *±* 0.001	0.081 *±* 0.007 ^a,b^	0.005 *±* 0.001	0.007 *±* 0.001
Sg L MH	0.042 *±* 0.003 ^a,b,c^	3.609 *±* 0.226 ^b^	0.006 *±* 0.002	0.072 *±* 0.004 ^c^	0.012 *±* 0.002	0.009 *±* 0.001 ^a^	0.004 *±* 0.001	0.102 *±* 0.006 ^a,b,c^	0.003 *±* 0.001	0.007 *±* 0.002
To F UAE	0.025 *±* 0.001 ^a^	3.115 *±* 0.198	0.015 *±* 0.002 ^a^	0.123 *±* 0.007 ^a,b^	0.009 *±* 0.001	0.005 *±* 0.001	0.004 *±* 0.001	0.063 *±* 0.003 ^a^	0.005 *±* 0.001	0.006 *±* 0.001
To F MH	0.037 *±* 0.002 ^a,b^	2.591 *±* 0.189 ^a,c^	0.011 *±* 0.002 ^a^	0.100 *±* 0.004 ^a,b,c^	0.010 *±* 0.002	0.006 *±* 0.000	0.004 *±* 0.000	0.070 *±* 0.006 ^a^	0.004 *±* 0.001	0.007 *±* 0.001
To L UAE	0.022 *±* 0.001 ^a,c^	3.420 *±* 0.220 ^b^	0.007 *±* 0.002	0.092 *±* 0.006 ^c^	0.011 *±* 0.001	0.005 *±* 0.002	0.003 *±* 0.000	0.079 *±* 0.004 ^a^	0.004 *±* 0.001	0.007 *±* 0.001
To L MH	0.038 *±* 0.002 ^a,b,c^	2.648 *±* 0.200 ^a,c^	0.012 *±* 0.002 ^a^	0.101 *±* 0.006 ^a,b,c^	0.008 *±* 0.001	0.007 *±* 0.001	0.003 *±* 0.001	0.065 *±* 0.005 ^a^	0.004 *±* 0.000	0.005 *±* 0.001
Tp F UAE	0.038 *±* 0.001 ^a,b,c^	3.780 *±* 0.182 ^b^	0.007 *±* 0.001	0.090 *±* 0.002 ^c^	0.010 *±* 0.002	0.005 *±* 0.001	0.003 *±* 0.000	0.106 *±* 0.005 ^a,b,c^	0.003 *±* 0.000	0.009 *±* 0.000 ^a^
Tp F MH	0.034 *±* 0.003 ^a,b^	2.451 *±* 0.176 ^a,c^	0.009 *±* 0.001	0.100 *±* 0.006 ^a,c^	0.006 *±* 0.000	0.006 *±* 0.001	0.003 *±* 0.000	0.077 *±* 0.005 ^a^	0.003 *±* 0.000	0.007 *±* 0.002
Ur L UAE	0.031 *±* 0.001 ^a,b^	3.125 *±* 0.198	0.014 *±* 0.002 ^a^	0.103 *±* 0.005 ^a,b,c^	0.008 *±* 0.000	0.008 *±* 0.002 ^a^	0.003 *±* 0.000	0.092 *±* 0.007 ^a,b^	0.005 *±* 0.001	0.008 *±* 0.000
Ur L MH	0.022 *±* 0.002 ^a,c^	3.399 *±* 0.218 ^b^	0.013 *±* 0.002 ^a^	0.117 *±* 0.005 ^a,b^	0.010 *±* 0.002	0.005 *±* 0.001	0.003 *±* 0.001	0.055 *±* 0.003 ^c^	0.005 *±* 0.001	0.004 *±* 0.001 ^c^
Vo R UAE	0.030 *±* 0.001 ^a,b^	3.600 *±* 0.210 ^b^	0.011 *±* 0.002 ^a^	0.069 *±* 0.004 ^c^	0.009 *±* 0.001	0.006 *±* 0.002	0.003 *±* 0.001	0.078 *±* 0.004 ^a^	0.007 *±* 0.001 ^c^	0.007 *±* 0.002
Vo R MH	0.020 *±* 0.001 ^a,c^	3.315 *±* 0.201 ^b^	0.010 *±* 0.000	0.120 *±* 0.006 ^a,b^	0.009 *±* 0.001	0.006 *±* 0.001	0.004 *±* 0.000	0.047 *±* 0.003 ^c^	0.005 *±* 0.000	0.003 *±* 0.001 ^c^
**Treatment**	**trans-geranylacetone**	**γ-curcumene**	**γ-gurjunene**	**β-ionone**	**β-selinene**	**α-cuprenene**	***E*** **-nerolidol**	**Longipinanol**	**3-butyl-hexahydro-phthalide**	**3-butylphthalide**
RT, min	41.555	43.050	43.230	43.380	43.790	45.010	48.100	48.840	51.865	52.810
C	0.003 *±* 0.001	0.043 *±* 0.004 ^b,c^	0.319 *±* 0.027 ^b,c^	0.063 *±* 0.006	0.054 *±* 0.002 ^b,c^	0.006 *±* 0.001	0.007 *±* 0.001	0.004 *±* 0.001 ^c^	0.051 *±* 0.001 ^c^	2.192 *±* 0.124
CF	0.002 *±* 0.000 ^c^	0.014 *±* 0.000 ^a^	0.576 *±* 0.006 ^a^	0.047 *±* 0.002	0.097 *±* 0.001 ^a^	0.004 *±* 0.001	0.006 *±* 0.001	0.005 *±* 0.001	0.077 *±* 0.003	1.997 *±* 0.023
CB	0.006 *±* 0.001 ^b^	0.012 *±* 0.003 ^a^	0.523 *±* 0.036 ^a^	0.056 *±* 0.006	0.092 *±* 0.005 ^a^	0.005 *±* 0.000	0.007 *±* 0.000	0.010 *±* 0.002 ^a^	0.093 *±* 0.007 ^a^	1.755 *±* 0.152
Hp H UAE	0.006 *±* 0.001 ^b^	0.034 *±* 0.004 ^b,c^	0.227 *±* 0.017 ^b,c^	0.060 *±* 0.005	0.039 *±* 0.002 ^a,b,c^	0.006 *±* 0.001	0.006 *±* 0.001	0.009 *±* 0.001 ^a^	0.094 *±* 0.007 ^a^	2.466 *±* 0.176 ^c^
Hp H MH	0.004 *±* 0.001	0.023 *±* 0.003 ^a^	0.297 *±* 0.020 ^b,c^	0.075 *±* 0.006 ^b^	0.052 *±* 0.003 ^b,c^	0.006 *±* 0.001	0.005 *±* 0.001	0.007 *±* 0.000	0.060 *±* 0.004 ^c^	2.119 *±* 0.152
Sg L UAE	0.004 *±* 0.001	0.012 *±* 0.002 ^a^	0.550 *±* 0.045 ^a^	0.039 *±* 0.005 ^a^	0.095 *±* 0.007 ^a^	0.005 *±* 0.000	0.005 *±* 0.000	0.009 *±* 0.001 ^a^	0.088 *±* 0.008 ^a^	1.784 *±* 0.170
Sg L MH	0.005 *±* 0.000	0.010 *±* 0.003 ^a^	0.334 *±* 0.022 ^b,c^	0.046 *±* 0.004	0.061 *±* 0.002 ^b,c^	0.004 *±* 0.001	0.004 *±* 0.001	0.009 *±* 0.001 ^a^	0.085 *±* 0.007 ^a^	1.269 *±* 0.115 ^a,b^
To F UAE	0.004 *±* 0.001	0.022 *±* 0.004 ^a^	0.399 *±* 0.027 ^b,c^	0.066 *±* 0.006	0.068 *±* 0.003 ^a,b,c^	0.004 *±* 0.001	0.005 *±* 0.000	0.005 *±* 0.001 ^c^	0.111 *±* 0.006 ^a,b^	2.428 *±* 0.143 ^c^
To F MH	0.004 *±* 0.001	0.013 *±* 0.002 ^a^	0.315 *±* 0.027 ^b,c^	0.054 *±* 0.005	0.056 *±* 0.004 ^b,c^	0.003 *±* 0.001	0.005 *±* 0.001	0.007 *±* 0.000	0.155 *±* 0.010 ^a,b,c^	1.931 *±* 0.178
To L UAE	0.002 *±* 0.000 ^c^	0.016 *±* 0.001 ^a^	0.365 *±* 0.029 ^b,c^	0.048 *±* 0.003	0.064 *±* 0.003 ^b,c^	0.005 *±* 0.001	0.003 *±* 0.000 ^a,c^	0.006 *±* 0.001	0.068 *±* 0.006	1.367 *±* 0.132 ^a,b^
To L MH	0.004 *±* 0.001	0.009 *±* 0.003 ^a^	0.347 *±* 0.031 ^b,c^	0.045 *±* 0.004	0.063 *±* 0.003 ^b,c^	0.003 *±* 0.000	0.004 *±* 0.000	0.006 *±* 0.001	0.095 *±* 0.003 ^a^	1.831 *±* 0.132
Tp F UAE	0.007 *±* 0.001 ^a,b^	0.015 *±* 0.002 ^a^	0.237 *±* 0.014 ^b,c^	0.056 *±* 0.005	0.047 *±* 0.002 ^b,c^	0.004 *±* 0.000	0.005 *±* 0.001	0.014 *±* 0.001 ^a,b^	0.238 *±* 0.012 ^a,b,c^	2.653 *±* 0.186 ^b,c^
Tp F MH	0.005 *±* 0.000	0.017 *±* 0.009 ^a^	0.516 *±* 0.064 ^a^	0.060 *±* 0.007	0.086 *±* 0.005 ^a^	0.003 *±* 0.000 ^a^	0.006 *±* 0.001	0.011 *±* 0.002 ^a,b^	0.215 *±* 0.007 ^a,b,c^	1.802 *±* 0.143
Ur L UAE	0.003 *±* 0.001	0.024 *±* 0.003 ^a^	0.455 *±* 0.030 ^a,b^	0.049 *±* 0.003	0.078 *±* 0.004 ^a,b,c^	0.006 *±* 0.000	0.006 *±* 0.001	0.019 *±* 0.002 ^a,b,c^	0.203 *±* 0.013 ^a,b,c^	2.041 *±* 0.144
Ur L MH	0.002 *±* 0.000 ^c^	0.025 *±* 0.003 ^a,c^	0.167 *±* 0.012 ^a,b,c^	0.069 *±* 0.006 ^b^	0.034 *±* 0.002 ^a,b,c^	0.005 *±* 0.001	0.004 *±* 0.001	0.007 *±* 0.000	0.130 *±* 0.010 ^a,b,c^	2.341 *±* 0.141 ^c^
Vo R UAE	0.005 *±* 0.001	0.020 *±* 0.002 ^a^	0.109 *±* 0.019 ^a,b,c^	0.055 *±* 0.008	0.023 *±* 0.004 ^a,b,c^	0.005 *±* 0.001	0.005 *±* 0.001	0.006 *±* 0.001	0.077 *±* 0.009	2.384 *±* 0.190 ^c^
Vo R MH	0.005 *±* 0.001	0.016 *±* 0.003 ^a^	0.528 *±* 0.037 ^a^	0.058 *±* 0.005	0.085 *±* 0.003 ^a^	0.005 *±* 0.000	0.005 *±* 0.000	0.004 *±* 0.000 ^c^	0.103 *±* 0.006 ^a^	1.993 *±* 0.157
**Treatment**	**3*Z*-butylidene phthalide**	**Unknown**	**Unknown**	**Senkyunolide**	**Neocnidilide**	***Z-l*** **igustilide**	***trans*** **-sedanolide**	***E*** **-α-atlantone**	**Neophytadiene**	**Nonadecane**
RT, min	53.580	53.775	54.085	54.935	55.035	55.185	55.375	56.035	56.840	57.615
C	0.021 *±* 0.002	0.230 *±* 0.038 ^c^	0.008 *±* 0.000	7.439 *±* 0.156 ^b,c^	1.276 *±* 0.036	0.033 *±* 0.005	0.125 *±* 0.008 ^b,c^	0.010 *±* 0.002	0.023 *±* 0.002 ^c^	0.083 *±* 0.006 ^b,c^
CF	0.016 *±* 0.000	0.184 *±* 0.002	0.007 *±* 0.001	4.971 *±* 0.055 ^a,c^	1.251 *±* 0.020	0.023 *±* 0.001	0.072 *±* 0.002 ^a^	0.010 *±* 0.001	0.037 *±* 0.001	0.021 *±* 0.001 ^a^
CB	0.017 *±* 0.003	0.125 *±* 0.016 ^a^	0.007 *±* 0.001	3.088 *±* 0.261 ^a,b^	1.294 *±* 0.093	0.017 *±* 0.002	0.066 *±* 0.006 ^a^	0.006 *±* 0.002	0.060 *±* 0.011 ^a^	0.019 *±* 0.006 ^a^
Hp H UAE	0.020 *±* 0.003	0.220 *±* 0.036 ^c^	0.008 *±* 0.001	5.470 *±* 0.249 ^a,c^	1.596 *±* 0.043 ^b^	0.030 *±* 0.006	0.067 *±* 0.006 ^a^	0.009 *±* 0.002	0.046 *±* 0.008	0.036 *±* 0.008 ^a^
Hp H MH	0.011 *±* 0.003	0.159 *±* 0.021	0.003 *±* 0.001 ^a^	4.549 *±* 0.266 ^a,c^	0.968 *±* 0.047 ^c^	0.018 *±* 0.004	0.031 *±* 0.003 ^a,b,c^	0.008 *±* 0.001	0.041 *±* 0.007	0.040 *±* 0.009 ^a^
Sg L UAE	0.011 *±* 0.002 ^a^	0.132 *±* 0.018 ^a^	0.004 *±* 0.000	3.251 *±* 0.249 ^a,b^	1.233 *±* 0.076	0.018 *±* 0.005	0.020 *±* 0.002 ^a,b,c^	0.005 *±* 0.001	0.059 *±* 0.008 ^a^	0.016 *±* 0.005 ^a^
Sg L MH	0.006 *±* 0.001 ^a,c^	0.076 *±* 0.010 ^a,b^	0.004 *±* 0.001	1.139 *±* 0.186 ^a,b,c^	0.795 *±* 0.086 ^a,b,c^	0.005 *±* 0.002	0.016 *±* 0.002 ^a,b,c^	0.004 *±* 0.000 ^b^	0.053 *±* 0.010	0.009 *±* 0.002 ^a^
To F UAE	0.020 *±* 0.002	0.210 *±* 0.026	0.010 *±* 0.001	4.643 *±* 0.172 ^a,c^	1.575 *±* 0.041 ^b^	0.031 *±* 0.005	0.039 *±* 0.002 ^a,b,c^	0.011 *±* 0.002	0.056 *±* 0.008 ^a^	0.036 *±* 0.009 ^a^
To F MH	0.015 *±* 0.002	0.154 *±* 0.022	0.007 *±* 0.001	3.363 *±* 0.176 ^a,b^	1.773 *±* 0.052 ^a,b,c^	0.020 *±* 0.006	0.024 *±* 0.002 ^a,b,c^	0.008 *±* 0.001	0.062 *±* 0.012 ^a^	0.021 *±* 0.006 ^a^
To L UAE	0.006 *±* 0.001 ^a,b,c^	0.110 *±* 0.018 ^a^	0.005 *±* 0.001	1.768 *±* 0.249 ^a,b,c^	0.773 *±* 0.096 ^a,b,c^	0.006 *±* 0.004	0.005 *±* 0.000 ^a,b,c^	0.004 *±* 0.001 ^a,b^	0.034 *±* 0.006	0.016 *±* 0.005 ^a^
To L MH	0.011 *±* 0.002 ^a^	0.129 *±* 0.016 ^a^	0.004 *±* 0.000	3.568 *±* 0.204 ^a,b^	1.289 *±* 0.053	0.019 *±* 0.004	0.017 *±* 0.002 ^a,b,c^	0.006 *±* 0.002	0.057 *±* 0.010 ^a^	0.017 *±* 0.004 ^a^
Tp F UAE	0.027 *±* 0.005 ^b^	0.130 *±* 0.019 ^a^	0.018 *±* 0.002 ^a,b,c^	3.616 *±* 0.241 ^a,b^	3.367 *±* 0.123 ^a,b,c^	0.023 *±* 0.005	0.132 *±* 0.011 ^b,c^	0.008 *±* 0.002	0.100 *±* 0.015 ^a,b,c^	0.025 *±* 0.005 ^a^
Tp F MH	0.017 *±* 0.003	0.115 *±* 0.015 ^a^	0.010 *±* 0.001	2.310 *±* 0.287 ^a,b^	1.973 *±* 0.218 ^a,b,c^	0.029 *±* 0.022	0.062 *±* 0.006 ^a^	0.006 *±* 0.002	0.039 *±* 0.008	0.011 *±* 0.003 ^a^
Ur L UAE	0.024 *±* 0.004	0.165 *±* 0.022	0.011 *±* 0.001	3.506 *±* 0.213 ^a,b^	1.772 *±* 0.064 ^a,b,c^	0.023 *±* 0.005	0.034 *±* 0.002 ^a,b,c^	0.008 *±* 0.002	0.065 *±* 0.011 ^a^	0.036 *±* 0.007 ^a^
Ur L MH	0.022 *±* 0.002	0.207 *±* 0.027	0.008 *±* 0.001	4.889 *±* 0.215 ^a,c^	1.675 *±* 0.027 ^a,b,c^	0.036 *±* 0.010	0.037 *±* 0.003 ^a,b,c^	0.011 *±* 0.000	0.038 *±* 0.006	0.029 *±* 0.006 ^a^
Vo R UAE	0.012 *±* 0.002	0.226 *±* 0.037 ^c^	0.004 *±* 0.001	5.811 *±* 0.408 ^a,c^	1.185 *±* 0.070	0.025 *±* 0.005	0.014 *±* 0.001 ^a,b,c^	0.013 *±* 0.001 ^c^	0.060 *±* 0.009 ^a^	0.048 *±* 0.006 ^a,b,c^
Vo R MH	0.019 *±* 0.003	0.159 *±* 0.023	0.006 *±* 0.001	3.501 *±* 0.253 ^a,b^	1.612 *±* 0.106 ^a,b^	0.023 *±* 0.007	0.028 *±* 0.002 ^a,b,c^	0.008 *±* 0.001	0.038 *±* 0.006	0.036 *±* 0.006 ^a^

(a) Statistically significant differences (*p* < 0.05) between the control group (C) and extracts. (b) Statistically significant differences (*p* < 0.05) between water formulation (CF) and extracts. (c) Statistically significant differences (*p* < 0.05) between commercial biostimulant (CB) and extracts. Abbreviations: RT, retention time; UAE, ultrasound assisted extraction; MH, mechanical homogenisation; Hp H, *Hypericum perforatum* L. (St. John’s wort, herb); Sg L, *Solidago gigantea* Ait. (giant goldenrod, leaf); To F, To L, *Taraxacum officinale* (L.) Weber ex F.H. Wigg (common dandelion, flower, leaf); Tp F, *Trifolium pratense* L. (red clover, flower); Ur L, *Urtica dioica* L. (nettle, leaf); Vo R, *Valeriana officinalis* L. (valerian, root).

**Table 6:** Effect of the foliar application of botanical extracts on the fatty acids composition (amount of single component calculated as percent (%) of whole GC-MS chromatogram area) of celeriac roots (N = 3, mean *±* SD).

**Treatment**	**Dodecanoic acid, methyl ester**	**Tridecanoic acid, 12-methyl-, methyl ester**	**Tetradecanoic acid, methyl ester**	**Tetradecanoic acid, ethyl ester**	**Tetradecanoic acid, 12-methyl-, methyl ester**	**Pentadecanoic acid, methyl ester**	***Z*** **-6-Octadecenoic acid, methyl ester**	**Pentadecanoic acid, 14-methyl-, methyl ester**	**Hexadecanoic acid, methyl ester**	***Z*** **-9-Hexadecenoic acid, methyl ester**	**Hexadecanoic acid, 15-methyl-, methyl ester**	**Hexadecanoic acid, 14-methyl-, methyl ester**
RT, min	18.875	23.000	24.285	25.665	26.110	26.910	27.405	28.245	29.455	30.235	30.725	31.155
C	0.90 *±* 0.09 ^b^	0.33 *±* 0.04	0.37 *±* 0.02 ^c^	2.01 *±* 0.09 ^c^	1.71 *±* 0.10 ^b,c^	0.82 *±* 0.06 ^c^	0.11 *±* 0.02	1.89 *±* 0.13 ^c^	25.69 *±* 0.13 ^b,c^	1.70 *±* 0.26 ^b^	2.22 *±* 0.18 ^b,c^	0.71 *±* 0.04 ^b,c^
CF	1.86 *±* 0.03 ^a,c^	0.44 *±* 0.02 ^c^	0.30 *±* 0.04	2.15 *±* 0.07 ^c^	2.20 *±* 0.04 ^a,c^	0.68 *±* 0.02	0.16 *±* 0.04	1.59 *±* 0.04 ^c^	21.07 *±* 0.11 ^a,c^	4.32 *±* 0.29 ^a,c^	0.87 *±* 0.08 ^a^	0.44 *±* 0.04 ^a^
CB	1.11 *±* 0.06 ^b^	0.24 *±* 0.02 ^b^	0.18 *±* 0.04 ^a^	1.35 *±* 0.01 ^a,b^	1.19 *±* 0.11 ^a,b^	0.67 *±* 0.02 ^a^	0.12 *±* 0.01	1.17 *±* 0.17 ^a,b^	23.32 *±* 0.13 ^a,b^	2.59 *±* 0.16 ^b^	0.73 *±* 0.06 ^a^	0.31 *±* 0.08 ^a^
Hp H UAE	1.07 *±* 0.06 ^b^	0.40 *±* 0.04 ^c^	0.20 *±* 0.07 ^a^	2.39 *±* 0.04 ^a,c^	2.24 *±* 0.10 ^a,c^	0.62 *±* 0.04 ^a^	0.10 *±* 0.04	2.10 *±* 0.02 ^b,c^	21.81 *±* 0.13 ^a,c^	2.48 *±* 0.31 ^b^	1.23 *±* 0.03 ^a,c^	0.66 *±* 0.10 ^c^
Hp H MH	1.23 *±* 0.08 ^b^	0.42 *±* 0.01 ^c^	0.29 *±* 0.02	2.37 *±* 0.04 ^a,c^	2.22 *±* 0.03 ^a,c^	0.71 *±* 0.04	0.14 *±* 0.02	1.71 *±* 0.11 ^c^	20.32 *±* 0.12 ^a,c^	2.54 *±* 0.47 ^b^	0.97 *±* 0.05 ^a^	0.55 *±* 0.01 ^c^
Sg L UAE	0.86 *±* 0.05 ^b^	0.49 *±* 0.01 ^a,c^	0.21 *±* 0.04 ^a^	2.85 *±* 0.02 ^a,b,c^	2.69 *±* 0.03 ^a,b,c^	0.65 *±* 0.00 ^a^	0.11 *±* 0.00	2.46 *±* 0.01 ^a,b,c^	22.12 *±* 0.22 ^a,b,c^	2.11 *±* 0.21 ^b^	1.43 *±* 0.04 ^a,b,c^	0.92 *±* 0.00 ^b,c^
Sg L MH	1.11 *±* 0.15 ^b^	0.37 *±* 0.01 ^c^	0.27 *±* 0.09	2.12 *±* 0.21 ^c^	1.97 *±* 0.25 ^c^	0.79 *±* 0.03	0.12 *±* 0.02	1.93 *±* 0.09 ^b,c^	22.34 *±* 0.10 ^a,b^	2.93 *±* 0.43 ^a,b^	0.94 *±* 0.14 ^a^	0.57 *±* 0.02 ^c^
To F UAE	0.80 *±* 0.07 ^b^	0.49 *±* 0.02 ^a,c^	0.23 *±* 0.02	2.04 *±* 0.03 ^c^	1.91 *±* 0.06 ^c^	0.78 *±* 0.08	0.04 *±* 0.02 ^b^	1.78 *±* 0.09 ^c^	23.28 *±* 0.47 ^a,b^	2.38 *±* 0.16 ^b^	1.33 *±* 0.09 ^a,c^	0.51 *±* 0.07
To F MH	1.57 *±* 0.07 ^a,c^	0.42 *±* 0.03 ^c^	0.28 *±* 0.03	2.09 *±* 0.01 ^c^	2.13 *±* 0.00 ^a,c^	0.73 *±* 0.03	0.15 *±* 0.00	1.65 *±* 0.02 ^c^	20.18 *±* 0.06 ^a,c^	3.81 *±* 0.18 ^a,c^	0.95 *±* 0.02 ^a^	0.52 *±* 0.03
To L UAE	1.23 *±* 0.27 ^b^	0.44 *±* 0.04 ^c^	0.40 *±* 0.06 ^c^	2.57 *±* 0.18 ^a,b,c^	2.67 *±* 0.27 ^a,b,c^	0.79 *±* 0.04	0.12 *±* 0.02	2.44 *±* 0.10 ^a,b,c^	22.85 *±* 0.64 ^a,b^	2.06 *±* 0.07 ^b^	1.20 *±* 0.41 ^a^	0.63 *±* 0.05 ^c^
To L MH	0.90 *±* 0.22 ^b^	0.45 *±* 0.02 ^c^	0.31 *±* 0.01	2.43 *±* 0.15 ^a,c^	2.24 *±* 0.05 ^a,c^	0.78 *±* 0.04	0.10 *±* 0.02	2.10 *±* 0.14 ^b,c^	22.76 *±* 0.49 ^a,b^	2.10 *±* 0.58 ^b^	1.19 *±* 0.17 ^a^	0.73 *±* 0.13 ^b,c^
Tp F UAE	1.33 *±* 0.07 ^a,b^	0.42 *±* 0.02 ^c^	0.30 *±* 0.01	2.25 *±* 0.07 ^c^	2.36 *±* 0.00 ^a,c^	0.71 *±* 0.03	0.15 *±* 0.01	1.83 *±* 0.06 ^c^	20.39 *±* 0.17 ^a,c^	3.25 *±* 0.09 ^a,b^	0.96 *±* 0.13 ^a^	0.53 *±* 0.09
Tp F MH	1.54 *±* 0.06 ^a,c^	0.63 *±* 0.06 ^a,b,c^	0.33 *±* 0.04	2.59 *±* 0.06 ^a,b,c^	2.70 *±* 0.02 ^a,b,c^	0.69 *±* 0.04	0.15 *±* 0.02	1.89 *±* 0.03 ^c^	18.09 *±* 0.30 ^a,b,c^	3.89 *±* 0.20 ^a,c^	0.93 *±* 0.03 ^a^	0.53 *±* 0.02
Ur L UAE	1.31 *±* 0.04 ^b^	0.36 *±* 0.02	0.30 *±* 0.01	2.25 *±* 0.03 ^c^	2.25 *±* 0.00 ^a,c^	0.61 *±* 0.03 ^a^	0.11 *±* 0.02	2.06 *±* 0.05 ^b,c^	20.76 *±* 0.05 ^a,c^	3.12 *±* 0.18 ^a,b^	1.33 *±* 0.01 ^a,c^	0.75 *±* 0.02 ^b,c^
Ur L MH	1.76 *±* 0.03 ^a,c^	0.59 *±* 0.08 ^a,b,c^	0.25 *±* 0.01	2.65 *±* 0.03 ^a,b,c^	2.66 *±* 0.09 ^a,b,c^	0.77 *±* 0.00	0.17 *±* 0.03	1.99 *±* 0.03 ^b,c^	19.33 *±* 0.09 ^a,b,c^	4.24 *±* 0.18 ^a,c^	0.92 *±* 0.02 ^a^	0.47 *±* 0.11 ^a^
Vo R UAE	2.01 *±* 0.12 ^a,c^	0.51 *±* 0.04 ^a,c^	0.36 *±* 0.01 ^c^	2.37 *±* 0.11 ^a,c^	2.34 *±* 0.05 ^a,c^	0.73 *±* 0.02	0.16 *±* 0.02	1.54 *±* 0.05 ^a,c^	16.51 *±* 0.28 ^a,b,c^	4.87 *±* 0.23 ^a,c^	0.92 *±* 0.10 ^a^	0.46 *±* 0.03 ^a^
Vo R MH	0.98 *±* 0.12 ^b^	0.33 *±* 0.03	0.25 *±* 0.00	1.87 *±* 0.01 ^c^	1.77 *±* 0.01 ^b,c^	0.70 *±* 0.03	0.10 *±* 0.01	1.82 *±* 0.02 ^c^	22.03 *±* 0.04 ^a,c^	2.50 *±* 0.11 ^b^	0.99 *±* 0.04 ^a^	0.58 *±* 0.02 ^c^
**Treatment**	**Heptadecanoic acid, methyl ester**	**Octadecanoic acid, methyl ester**	***9Z-*** **9-Octadecenoic acid, ethyl ester**	**11-Octadecenoic acid, methyl ester**	**9, 12-Hexadecadienoic acid, methyl ester**	***Z, Z, Z*** **-9, 12, 15-Octadecatrienoic acid, methyl ester**	**Methyl 18-methylnonadecanoate**	***cis*** **-Methyl 11-eicosenoate**	**Docosanoic acid, methyl ester**	**Tricosanoic acid, methyl ester**	**Tetracosanoic acid, methyl ester**	**Tetracosanoic acid, ethyl ester**
RT, min	31.885	34.245	34.94	35.125	36.335	37.745	38.14	38.71	40.715	41.4	41.59	42.69
C	0.30 *±* 0.06 ^b^	3.82 *±* 0.22 ^b,c^	3.17 *±* 0.17	0.71 *±* 0.02 ^c^	43.46 *±* 1.17 ^b,c^	4.28 *±* 0.09 ^b,c^	0.60 *±* 0.03 ^b,c^	0.18 *±* 0.03 ^b^	1.05 *±* 0.20 ^b,c^	1.53 *±* 0.13 ^c^	1.31 *±* 0.13 ^b,c^	0.94 *±* 0.06 ^b,c^
CF	0.16 *±* 0.01 ^a^	1.75 *±* 0.11 ^a,c^	3.80 *±* 0.13 ^c^	0.61 *±* 0.05	48.76 *±* 0.75 ^a,c^	5.33 *±* 0.08 ^a^	0.18 *±* 0.04 ^a^	0.39 *±* 0.02 ^a^	0.33 *±* 0.05 ^a^	1.58 *±* 0.17 ^c^	0.73 *±* 0.08 ^a,c^	0.26 *±* 0.03 ^a^
CB	0.24 *±* 0.01	2.44 *±* 0.07 ^a,b^	2.55 *±* 0.13 ^b^	0.44 *±* 0.05 ^a^	53.90 *±* 0.32 ^a,b^	5.18 *±* 0.05 ^a^	0.29 *±* 0.03 ^a^	0.26 *±* 0.00	0.38 *±* 0.05 ^a^	0.62 *±* 0.11 ^a,b^	0.39 *±* 0.06 ^a,b^	0.32 *±* 0.06 ^a^
Hp H UAE	0.23 *±* 0.01	1.90 *±* 0.01 ^a,c^	3.95 *±* 0.35 ^a,c^	0.73 *±* 0.06 ^c^	50.68 *±* 0.38 ^a,c^	4.11 *±* 0.02 ^b,c^	0.43 *±* 0.05 ^a,b^	0.45 *±* 0.01 ^a,c^	0.41 *±* 0.05 ^a^	0.97 *±* 0.06 ^a,b^	0.51 *±* 0.06 ^a^	0.30 *±* 0.02 ^a^
Hp H MH	0.19 *±* 0.00	1.32 *±* 0.03 ^a,c^	3.21 *±* 0.09	0.50 *±* 0.00 ^a^	52.84 *±* 0.62 ^a,b^	5.38 *±* 0.12 ^a^	0.23 *±* 0.02 ^a^	0.28 *±* 0.01	0.30 *±* 0.06 ^a^	1.23 *±* 0.10 ^c^	0.75 *±* 0.01 ^a,c^	0.27 *±* 0.05 ^a^
Sg L UAE	0.27 *±* 0.02	2.30 *±* 0.05 ^a,b^	2.53 *±* 0.21 ^b^	0.59 *±* 0.04	50.21 *±* 0.65 ^a,c^	4.21 *±* 0.03 ^b,c^	0.41 *±* 0.03 ^a,b^	0.22 *±* 0.04	0.56 *±* 0.10 ^a^	0.88 *±* 0.11 ^a,b^	0.50 *±* 0.06 ^a,b^	0.39 *±* 0.05 ^a^
Sg L MH	0.24 *±* 0.02	1.80 *±* 0.27 ^a,c^	3.13 *±* 0.06	0.60 *±* 0.01	51.03 *±* 1.08 ^a,c^	4.73 *±* 0.17 ^a,b,c^	0.33 *±* 0.06 ^a,b^	0.52 *±* 0.11 ^a,c^	0.36 *±* 0.05 ^a^	0.80 *±* 0.07 ^a,b^	0.70 *±* 0.08 ^a,c^	0.28 *±* 0.04 ^a^
To F UAE	0.28 *±* 0.05 ^b^	2.21 *±* 0.04 ^a^	2.55 *±* 0.11 ^b^	0.44 *±* 0.09 ^a^	51.97 *±* 0.47 ^a,b^	4.55 *±* 0.22 ^b,c^	0.30 *±* 0.03 ^a^	0.33 *±* 0.08	0.41 *±* 0.02 ^a^	0.69 *±* 0.04 ^a,b^	0.46 *±* 0.02 ^a,b^	0.24 *±* 0.02 ^a^
To F MH	0.14 *±* 0.04 ^a^	1.11 *±* 0.02 ^a,b,c^	2.85 *±* 0.07 ^b^	0.46 *±* 0.07 ^a^	52.67 *±* 0.34 ^a,b^	5.15 *±* 0.02 ^a^	0.24 *±* 0.03 ^a^	0.33 *±* 0.02	0.27 *±* 0.06 ^a^	1.36 *±* 0.10 ^c^	0.66 *±* 0.04 ^a,c^	0.23 *±* 0.03 ^a^
To L UAE	0.32 *±* 0.03 ^b^	1.69 *±* 0.16 ^a,c^	2.44 *±* 0.31 ^b^	0.46 *±* 0.08 ^a^	50.66 *±* 1.12 ^a,c^	4.87 *±* 0.28 ^a,b^	0.17 *±* 0.03 ^a^	0.32 *±* 0.08	0.36 *±* 0.01 ^a^	0.72 *±* 0.03 ^a,b^	0.32 *±* 0.02 ^a,b^	0.16 *±* 0.05 ^a^
To L MH	0.26 *±* 0.03	2.25 *±* 0.35 ^a,b^	4.55 *±* 0.40 ^a,b,c^	0.63 *±* 0.05	49.10 *±* 1.33 ^a,c^	4.62 *±* 0.14 ^b,c^	0.34 *±* 0.03 ^a,b^	0.31 *±* 0.05	0.38 *±* 0.06 ^a^	0.91 *±* 0.04 ^a,b^	0.61 *±* 0.05 ^a^	0.26 *±* 0.06 ^a^
Tp F UAE	0.13 *±* 0.04 ^a,c^	1.73 *±* 0.06 ^a,c^	2.94 *±* 0.11 ^b^	0.44 *±* 0.08 ^a^	52.14 *±* 0.61 ^a,b^	5.23 *±* 0.11 ^a^	0.19 *±* 0.02 ^a^	0.41 *±* 0.05 ^a^	0.28 *±* 0.04 ^a^	1.18 *±* 0.02 ^b,c^	0.57 *±* 0.07 ^a^	0.22 *±* 0.06 ^a^
Tp F MH	0.14 *±* 0.01 ^a^	0.93 *±* 0.06 ^a,b,c^	2.16 *±* 0.07 ^a,b^	0.44 *±* 0.04 ^a^	52.84 *±* 0.43 ^a,b^	6.07 *±* 0.07 ^a,b,c^	0.20 *±* 0.06 ^a^	0.33 *±* 0.01	0.32 *±* 0.06 ^a^	1.45 *±* 0.19 ^c^	0.84 *±* 0.07 ^a,c^	0.26 *±* 0.01 ^a^
Ur L UAE	0.27 *±* 0.02 ^b^	1.92 *±* 0.03 ^a,c^	2.88 *±* 0.37 ^b^	0.63 *±* 0.02	51.09 *±* 0.26 ^a,c^	4.52 *±* 0.04 ^b,c^	0.41 *±* 0.00 ^a,b^	0.40 *±* 0.01 ^a^	0.48 *±* 0.02 ^a^	1.06 *±* 0.07 ^a,b,c^	0.52 *±* 0.02 ^a^	0.60 *±* 0.07 ^a,b,c^
Ur L MH	0.18 *±* 0.01 ^a^	1.01 *±* 0.03 ^a,b,c^	2.32 *±* 0.02 ^a,b^	0.43 *±* 0.05 ^a^	51.81 *±* 0.16 ^a,b^	5.50 *±* 0.12 ^a^	0.22 *±* 0.04 ^a^	0.32 *±* 0.07	0.32 *±* 0.03 ^a^	0.91 *±* 0.08 ^a,b^	0.78 *±* 0.05 ^a,c^	0.34 *±* 0.03 ^a^
Vo R UAE	0.13 *±* 0.03 ^a,c^	0.87 *±* 0.03 ^a,b,c^	2.49 *±* 0.08 ^b^	0.46 *±* 0.04 ^a^	52.53 *±* 0.63 ^a,b^	6.74 *±* 0.06 ^a,b,c^	0.34 *±* 0.08 ^a,b^	0.67 *±* 0.06 ^a,b,c^	0.41 *±* 0.06 ^a^	1.32 *±* 0.12 ^c^	0.83 *±* 0.06 ^a,c^	0.36 *±* 0.03 ^a^
VoR MH	0.29 *±* 0.00 ^b^	2.17 *±* 0.03 ^a^	3.10 *±* 0.06	0.65 *±* 0.03 ^c^	52.01 *±* 0.45 ^a,b^	4.86 *±* 0.05 ^a,b^	0.45 *±* 0.03 ^a,b,c^	0.38 *±* 0.02 ^a^	0.51 *±* 0.03 ^a^	0.84 *±* 0.03 ^a,b^	0.48 *±* 0.02 ^a,b^	0.34 *±* 0.02 ^a^

(a) Statistically significant differences (*p* < 0.05) between the control group (C) and extracts. (b) Statistically significant differences (*p* < 0.05) between water formulation (CF) and extracts. (c) Statistically significant differences (*p* < 0.05) between commercial biostimulant (CB) and extracts. Abbreviations: RT, retention time; UAE, ultrasound assisted extraction; MH, mechanical homogenisation; Hp H, *Hypericum perforatum* L. (St. John’s wort, herb); Sg L, *Solidago gigantea* Ait. (giant goldenrod, leaf); To F, To L, *Taraxacum officinale* (L.) Weber ex F.H. Wigg (common dandelion, flower, leaf); Tp F, *Trifolium pratense* L. (red clover, flower); Ur L, *Urtica dioica* L. (nettle, leaf); Vo R, *Valeriana officinalis* L. (valerian, root).

**Table 7:** Natural raw materials used for biostimulant production and their application in plant cultivation.

Source of Bioactive Compounds	Application Method	Applied Concentration, %	Plant Species	Test Type	Effect on Plants	Ref.
–moringa (*Moringa oleifera*) (leaves)	foliar spray	5, 10, 20, 30	coriander (*Coriandrum sativum* var. *microcarpum*)	field	increased fruit yield, volatile oil yield, oil components, percentages of N, P, K, total sugars, the radical scavenging activity and total phenolic content	[58]
–moringa (*Moringa oleifera*) (leaves)	foliar spray	3	Sudan grass (*Sorghum vulgare* var. *sudanense*)	pot (an open glasshouse)	under saline conditions –increased growth characteristics, photochemical activity, contents of RNA, DNA, phytohormones, osmoprotectants and non-enzymatic antioxidants, and activities of antioxidant enzymes	[59]
–moringa (*Moringa oleifera*) (leaves)	foliar spray	3	quinoa (*Chenopodium quinoa*)	pot (a wire house)	mitigated adverse effects of heat stress; increased photosynthetic rate, intrinsic water use efficiency; improved leaf chlorophyll and antioxidants; increased seed yield under normal and heat stress conditions	[60]
–moringa (*Moringa oleifera*) (leaves)	foliar spray	4, 5, 6	five years old of ‘Hollywood’ plum trees (*Prunus salicina*)	orchard	increased setting, yield, fruit weight, firmness, colour, soluble solid content, titratable acidity ratio, ascorbic acid, anthocyanin content, antioxidant activity contents; reduced titratable acidity with reduced fruit drop	[61]
–moringa (*Moringa oleifera*, *M. peregrena*) (leaves)	irrigation	2.5, 5, 10, 20	sweet basil (*Ocimum basilicum* cv. Cispum)	pot (a glasshouse)	decreased proline and malondialdehyde during salt stress; enlarged leaf area; increased shoot length, shoot fresh weight, shoot dry weight, number of branches, root length, root dry weight, anthocyanin, total carbohydrates and superoxide dismutase, ascorbic acid oxidase	[62]
–moringa (*Moringa oleifera*)	priming, foliar spray	3	wheat (*Triticum aestivum* cv. Galaxy)	pot (a wire house)	improved speed and spread of emergence, seedling vigour (shoot and root length, fresh and dry weight), final emergence count; reduced time to 50% emergence, mean emergence time; improved emergence index	[63]
–red grape (*Vitis vinifera*) (skin) –blueberry (*Vaccinium vitis-idaea*) (fruits) –hawthorn (*Crataegus monogina*) (leaves)	hydroponic solution (48 h)	0.01, 0.1	maize (*Zea mays*)	pot (an aerated complete culture solution; a climatic chamber)	increased root and leaf biomass, chlorophyll, phenol acids and sugars content; induced phenylalanine ammonia lyase (PAL) activity	[64]
–black elder (*Sambucus nigra* (flowers) –silver birch (*Betula verrucosa*) (leaves) –mugwort (*Artemisia sativum*) (bulb) –garlic (*Allium sativum*) (bulb) –horse chestnut (*Aesculus hippocastanum*) (bark, flowers) –peppermint (*Mentha piperita*) (leaves) –common soapwort (*Saponaria officinalis*) (root) –nettle (*Urtica dioica*) (leaves) –field horsetail (*Equisetum arvense*) (herb) –horehound (*Marrubium vulgare*) (herb) –sweet flag (*Acorus calamus*) (rhizome) –hawthorn (*Crataegus oxyacantha*) (flower) –alder buckthorn (*Frangula alnus*) (bark) –maize (*Zea mays*) (moles) –lemon balm (*Melissa officinalis*) (leaves) –common dandelion (*Taraxacum officinale*) (root) –elecampane (*Inula helenium*) (root) –chamomile (*Matricaria chamomilla*) (baskets) –dog rose (*Rosa canina*) (fruit)	seed dressing	100	cauliflower (*Brassica oleracea* var. *botrytis*)	germination	stimulated seed germination; reduced seed infestation by pathogenic microorganisms	[65]
–bee-honey	foliar spray	2.5, 5	onion (*Allium cepa*)	field	increased biomass production, bulb yield, water use efficiency, leaf photosynthetic and pigments contents; improved osmoprotectants, membrane stability index, relative water content, enzymatic and non-enzymatic antioxidants	[66]
–garlic (*Allium sativum*) (cloves)	foliar spray	2.44, 4.76, 9.09	snap bean (*Phaseolus vulgaris* cv. Paulista)	field	enhanced height, leaf area, leaves number, plant weight, flowers number, leaf and pod chemical compositions; increase in number of pods, pod fresh weight, total pod yield	[67]
–garlic (*Allium sativum*) (bulb)	foliar spray, fertigation	0.01	eggplant (*Solanum melongena*) and pepper (*Capsicum*)	pot (a glasshouse)	improved plant height, number of leaves, root growth, fresh and dry weight; indicated alterations in metabolites (chlorophyll, carotenoids, soluble sugars); stimulated antioxidant enzymes (superoxide dismutase, peroxidase), root activity; induced defence responses prior to *Phytopthora capsica* inoculation	[68]
–olive (*Olea europaea*) (leaves) –pomegranate (*Punica granatum*) (leaves) –common guava (*Psidium guajava*) (leaves)	foliar spray	0.5	sesame (*Sesamum indicum*) (Shandawil 3)	field	increased carotenoids, total carbohydrates and lipids content, amylase and peroxidase activities; exerted a varied influence on fresh and dry weight of shoots, fresh weight of roots, shoots and roots length, number and weight of pods and seeds per plant, number of leaves, content of chlorophyll, content of carbohydrates in shoots, content of proteins in roots and shoots; decreased dry weight of roots, protease and catalase activities, total proteins content	[69]
–garlic (*Allium sativum*) (bulb)	foliar spray	0.02	eggplant (*Solanum melongena*)	plastic tunnel	–a one pre-transplant spraying –improved growth, plant morphology and biomass, enhanced antioxidant enzymes (superoxide dismutase, peroxidase), photosynthesis and chlorophyll content; –triple application –inhibited plant growth and development and resulted in lipid peroxidation (increased content of malondialdehyde); –post-transplant application –increased growth, did not exert significant increase in the malondialdehyde content	[70]
–sugar beet (*Beta vulgaris*)	seed priming/soaking (12 h)	10, 20, 30, 40, 50	wheat (*Triticum aestivum*)	germination and pot (natural environmental conditions)	–under water stress –ameliorated germination attributes (time to 50% emergence, germination index, mean emergence time, germination percentage, coefficient of uniformity of emergence, germination energy); –with and without water stress –improved plant growth, photosynthetic pigments, antioxidants’ activities and nutrient homeostasis	[71]
–lantana (*Lantana camara*)	spray	0.5, 1	green gram (*Vigna radiata*)	pot (a glasshouse)	increased plant height, number of leaves, dry matter, chlorophyll content, number and weight of pods per plant, number of seeds per pod, grain yield	[72]
–liquorice (*Glycyrrhiza glabra*) (root)	rhizosphere application, foliar spray	0.5	pepper (*Capsicum annuum*)	field	increased plant growth and yield, concentrations of photosynthetic pigments, free proline, total soluble sugars, N, P, and K^+^, ratio of K^+^/Na^+^, activities of catalase, peroxidase, ascorbate peroxidase, superoxide dismutase and glutathione reductase; reduced contaminants; Na, Cd, Cu, Pb and Ni concentrations in leaves and fruits on heavy metals-contaminated saline soil	[73]
–liquorice (*Glycyrrhiza glabra*) (root)	seed soaking, foliar spray	0.5	common bean (*Phaseolus vulgaris* cv. Bronco)	field	–preliminary study under salt stress –increased plant growth, yield, relative water content, chlorophylls content –preliminary study without salt stress –aforementioned parameters were not altered –field studies under salt stress –increased growth and yield parameters, photosynthetic pigments, free proline, total soluble carbohydrates, total soluble sugars, nutrients, and selenium, ratio of K^+^/Na^+^, relative water content, membrane stability index, activities of enzymatic antioxidants, anatomical features; decreased electrolyte leakage, malondialdehyde, Na^+^, hydrogen peroxide, superoxide radical	[74]
–palm pollen (*Phoenix dactylifera*) (grains)	foliar spray	0.1	sweet basil (*Ocimum basilicum*)	pot (an open glasshouse)	under drought stress –improved growth characteristics and the contents of essential oil, leaf photosynthetic pigments, soluble sugars, free proline, ascorbic acid, antioxidant enzyme activities, relative water content, water use efficiency, and anatomical characteristics; diminished electrolyte leakage	[75]
commercial biostimulants: –legume-derived protein hydrolysate-LDPH (Trainer^®^) –tropical plant extract-TPE (Auxym^®^)	foliar spray	0.3 (LDPH), 0.2 (TPE)	baby rocket leaf (*Diplotaxis erucoides* cv. Reset)	lysimeters (an open plastic tunnel)	enhanced plant growth, increased marketable yield, the SPAD index, ascorbic acid and leaf pigments content (chlorophyll, carotenoids), lipophilic antioxidant activity	[32]
commercial biostimulant: –legume-derived protein hydrolysate Trainer^®^	foliar spray	0.3	baby spinach (*Spinacia oleracea*, cv. Donkey F1)	glasshouse	enhanced fresh yield, dry biomass, leaf area, chlorophyll biosynthesis, bioactive compounds (total phenols, ascorbic acid), potassium and magnesium concentrations; did not exert significant increase in nitrate accumulation	[27]
–borage (*Borago officinalis*) (leaves, flowers)	foliar spray	0.1, 1	lettuce (*Lactuca sativa*)	pot (a glasshouse)	enhanced primary metabolism, increased leaf pigments and photosynthetic activity, plant fresh weight, chlorophyll *a* fluorescence, total flavonoids and phenols, total protein levels, in vitro PAL specific activity, and the levels of PAL-like polypeptides; prevented degradation and induced increase in photosynthetic pigments during storage decreased ethylene content; did not exert significant impact on nitrate and sugar levels	[76]
–cultivated tobacco (*Nicotiana tabacum*) (leaves) –bael (*Aegle marmelos*) (leaves) –fig tree (*Ficus hispida*) (leaves) –hina (*Lawsonia inermis*) (leaves) –Chinese chaste tree (*Vitex negundo*) (leaves) –wild celery (*Carum roxburghianum*) (seeds) –white jute (*Corchorus capsularis*) (seeds) –mahogany (*Swietenia macrophylla*) (seeds) –garlic (*Allium sativum*) (bulb)	spray	100	eggplant (*Solanum melongena*)	field	–*Nicotiana tabacum* leaves extract –increased resistance against pest attack; enhanced growth, yield and longevity of plant life –*Allium sativum* bulb extract –showed very poor efficacy to protect leaves from pest attack caused total inhibition of fruit production –*Swietenia macrophylla* and *Carum roxburghianum* seeds extracts –showed phyto-toxicity and hampered the growth –*Carum roxburghianum* –caused total inhibition of fruit production	[77]
–amazon cinnamon (*Ocotea quixos*) (leaves) –pepper (*Piper carpunya*) (plant material)	spray	40, 60	red ginger (*Alpinia purpurata*), heliconia (*Heliconia wagneriana*)	field	showed an inhibitory activity against *Fusarium* sp. and *Capnodium* sp., biostimulating effect on flower spikes	[78]
–French oak (*Quercus sessiliflora*) (chips)	foliar spray	25, 100	8-year-old grapevines (*Vitis vinifera* var. Monastrell)	vineyard	affected composition, producing less alcoholic and acid wines with higher colour intensity, lower shade, more stable colour, higher content of polyphenols	[79]
–alfalfa plant (*Medicago sativa*)	supplement to the culture solution (48 h)	0.0001	maize (*Zea mays*)	pot (an aerated complete culture solution a climate chamber)	stimulated the growth and nitrogen assimilation in conditions with and without drought stress increased biomass, gene expression and decreased the activity of antioxidant enzymes and the synthesis of phenolics induced the activity of enzymes functioning in nitrogen metabolism enhanced PAL activity and flavonoids content during stress	[80]
–carrot (*Daucus carota*) (roots)	seed pre-soaking	25, 50	cowpea (*Vigna sinensis*)	germination	increased growth parameters, total chlorophyll, carotenoids, total carbohydrate, antioxidant compounds content during salt stress—showed differential expression of the genetic information (changes in gene products, including protein and isozymes profiles)	[81]
–apple (seeds) –colza (seeds) –rice (husks)	foliar spray	not available	kiwifruit (*Actinidia deliciosa* cv. Hayward and cv. Green Light)	orchard	increased the fruit weight, ascorbic acid content, dry matter, antioxidant capacity	[82]

## Supplementary Materials

The following are available online. Figure S1: The weather conditions during the field experiments.
