# Supplementary figures and images for: Field-Scale Evaluation of Botanical Extracts Effect on the Yield, Chemical Composition and Antioxidant Activity of Celeriac (Apium graveolens L. Var. rapaceum)
Source: Molecules. 2020 Sep 14;25(18):4212. doi: 10.3390/molecules25184212 (PMC7571039; doi:10.3390/molecules25184212)

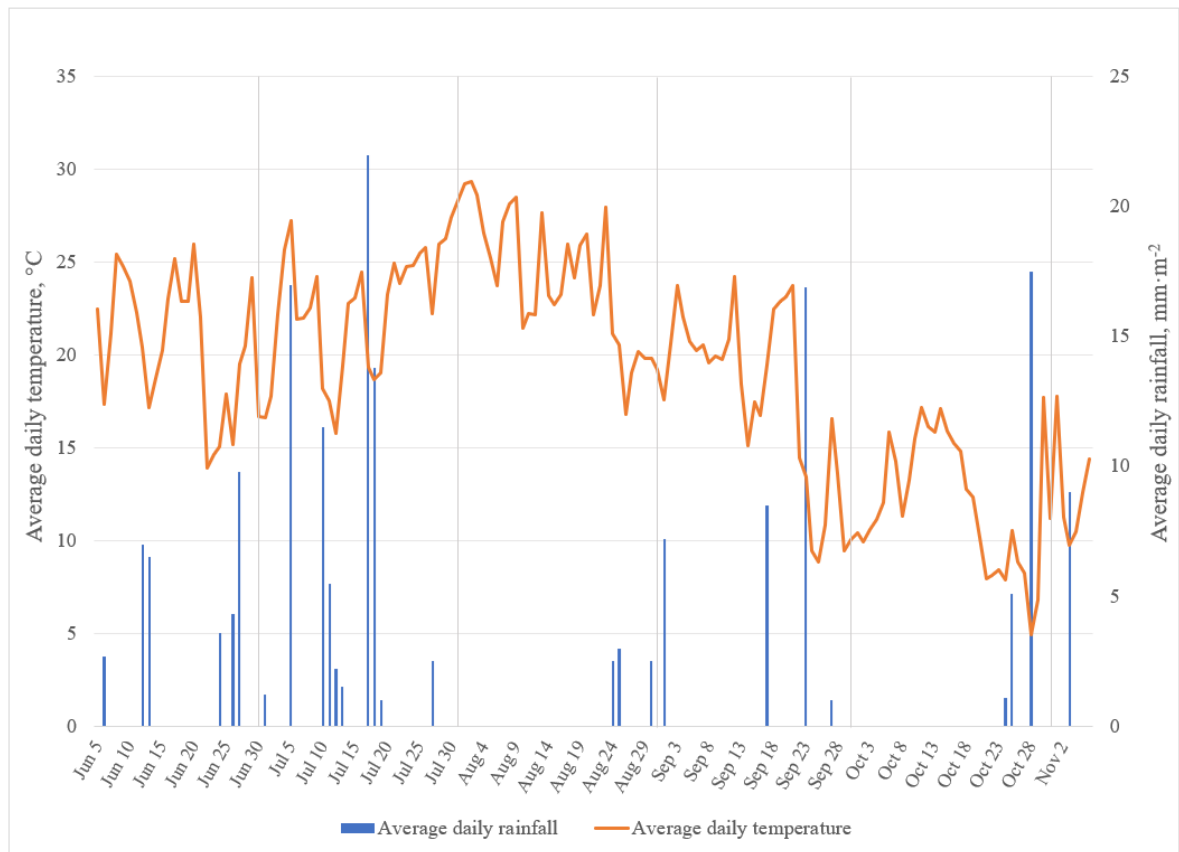

**Figure S1.** The weather conditions during the field experiments.

Supplement: Supplementary file 1 [file molecules-25-04212-s001.pdf]
